# On the Embryonic Development of the Nasal Turbinals and Their Homology in Bats

**DOI:** 10.3389/fcell.2021.613545

**Published:** 2021-03-23

**Authors:** Kai Ito, Vuong Tan Tu, Thomas P. Eiting, Taro Nojiri, Daisuke Koyabu

**Affiliations:** ^1^Department of Anatomy, Tissue and Cell Biology, School of Dental Medicine, Tsurumi University, Yokohama, Japan; ^2^Institute of Ecology and Biological Resources, Vietnam Academy of Science and Technology, Hanoi, Vietnam; ^3^Graduate University of Science and Technology, Vietnam Academy of Science and Technology, Hanoi, Vietnam; ^4^Department of Neurobiology and Anatomy, University of Utah, Salt Lake City, UT, United States; ^5^Graduate School of Agricultural and Life Sciences, The University of Tokyo, Tokyo, Japan; ^6^The University Museum, The University of Tokyo, Tokyo, Japan; ^7^Research and Development Center for Precision Medicine, University of Tsukuba, Tsukuba, Japan; ^8^Jockey Club College of Veterinary Medicine and Life Sciences, City University of Hong Kong, Kowloon, Hong Kong; ^9^Department of Molecular Craniofacial Embryology, Tokyo Medical and Dental University, Tokyo, Japan

**Keywords:** Chiroptera, evo-devo, skull, microCT (μCT), homology

## Abstract

Multiple corrugated cartilaginous structures are formed within the mammalian nasal capsule, eventually developing into turbinals. Due to its complex and derived morphology, the homologies of the bat nasal turbinals have been highly disputed and uncertain. Tracing prenatal development has been proven to provide a means to resolve homological problems. To elucidate bat turbinate homology, we conducted the most comprehensive study to date on prenatal development of the nasal capsule. Using diffusible iodine-based contrast-enhanced computed tomography (diceCT), we studied in detail the 3D prenatal development of various bat species and non-bat laurasiatherians. We found that the structure previously identified as “maxilloturbinal” is not the true maxilloturbinal and is only part of the ethmoturbinal I pars anterior. Our results also allowed us to trace the evolutionary history of the nasal turbinals in bats. The turbinate structures are overall comparable between laurasiatherians and pteropodids, suggesting that pteropodids retain the ancestral laurasiatherian condition. The absence of the ethmoturbinal I pars posterior in yangochiropterans and rhinolophoids has possibly occurred independently by convergent evolution.

## Introduction

The mammalian nasal cavity contains a series of bony and cartilaginous plate-like structures called turbinals, which together project into the nasal cavity and provide surface area for various functions (Moore, 1981; Van Valkenburgh et al., 2014; Smith et al., 2015). Generally, the roles of the nasal cavity are twofold: to heat and humidify inhaled air before entering the lungs and to provide surface area for odorant deposition and olfactory sensation (Negus, 1958; Hillenius, 1992). The turbinals projecting into the nasal cavity primarily provide a surface area, offering a scaffold for blood vessels, secretory cells, and olfactory cells (Negus, 1958; Moore, 1981; Smith and Rossie, 2008; Van Valkenburgh et al., 2011; Wagner and Ruf, 2019, 2020).

Several types of turbinal can be recognized in the mammalian nasal cavity. The marginoturbinal and atrioturbinal are found in the outer nasal cartilage in the rostral part of the nasal cavity. The marginoturbinal begins at the lateral margin of the external nasal opening and continues into the atrioturbinal (Maier, 1980, 2000). The shape of these turbinals forms the naris and permits effective airflow (Göbbel, 2000; Maier and Ruf, 2014). These turbinals remain cartilaginous in adults and are continuous with the maxilloturbinal (Voit, 1909; Reinbach, 1952a, b; Maier, 1980, 1993b; Zeller, 1987; Smith et al., 2015). The maxilloturbinal is ventrally positioned in the nasal cavity (Negus, 1958; Moore, 1981; Smith et al., 2015). This turbinal projects from the medial surface of the maxilla and is covered with the respiratory epithelium (Scott, 1954; Adams, 1972; Van Valkenburgh et al., 2011) to add humidity to and increase the temperature of inhaled air (Scott, 1954). The maxilloturbinal generally becomes the largest and most complex in adults (Maier, 1993b; Maier and Ruf, 2014; Van Valkenburgh et al., 2014; Smith et al., 2015). The nasoturbinal projects from the roof of the nasal cavity (Moore, 1981). This turbinal articulates with the inferior margin of the nasal bone and medial surface of the maxilla and extends caudally into the ethmoid complex (Moore, 1981; Smith et al., 2015). The ethmoturbinals project from the lateral mass of the ethmoid bone (Smith et al., 2015). Several ethmoturbinals are found (Van Gilse, 1927; Maier, 1993a; Maier and Ruf, 2014) and are generally covered with olfactory epithelium (Adams, 1972; Gross et al., 1982; Martinez et al., 2020). Each ethmoturbinal is arranged one behind the other in parallel (Voit, 1909). Voit (1909) denoted the ethmoturbinals by Roman numerals in rostrocaudal sequence. Ethmoturbinal I protrudes toward the nostrils and is usually the largest among the ethmoturbinals (Voit, 1909). It makes the front border of the ethmoturbinal recess, which is the restricted space in the caudal part within the nasal cavity (Smith and Rossie, 2008; Maier and Ruf, 2014). The number of ethmoturbinals varies among species (Paulli, 1900a,b,c; Rowe et al., 2005). To our knowledge, the minimum number is seen in *Tursiops* in odontocetes with the absence of the ethmoturbinal (Mead and Fordyce, 2009). *Orycteropus afer* has the maximum number of ethmoturbinal so far with “at least nine” (Stößel et al., 2010). The frontoturbinals are located within the frontoturbinal recess, which is the dorsocaudal space of the lateral recess bounded ventrally by the root of ethmoturbinal I (Maier, 1993a; Rossie, 2006). The accessory scrolls between the frontoturbinals within the frontoturbinal recess are known as interturbinals (Maier, 1993b; Maier and Ruf, 2014; Ruf, 2014). The number of frontoturbinals and interturbinals may vary depending on the species (Smith et al., 2015).

In addition to turbinals, the nasal cavity presents other sheet-like ossifications such as the lamina semicircularis, lamina horizontalis, and lamina transversalis (Maier and Ruf, 2014). The lamina semicircularis is the medial wall of the maxillary recess and frontoturbinal recess (Ruf, 2014). This lamina is continuous with the posterior part of the nasoturbinal (Macrini, 2012; Smith et al., 2015). The lamina horizontalis separates the lateral recess into the dorsal and ventral chambers: the dorsal chamber is the frontoturbinal recess and the ventral chamber is the maxillary recess (Smith and Rossie, 2008; Maier and Ruf, 2014). The lamina transversalis extends from the lateral walls of the nasal cavity and attaches to the nasal septum, separating the ethmoturbinal recess from the nasopharyngeal duct (Lozanoff and Diewert, 1989; Macrini, 2012; Smith et al., 2015).

**FIGURE 1:**
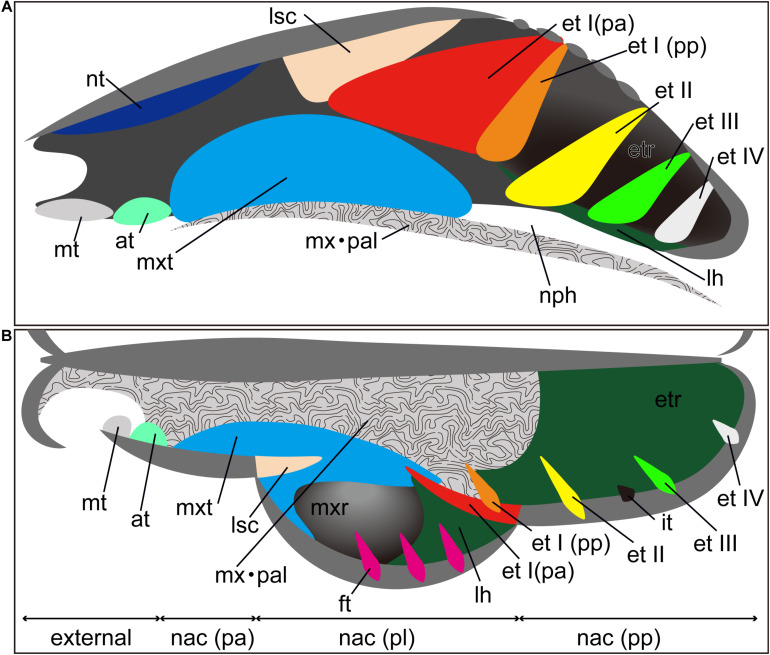
Generalized schematic mammalian nasal capsule (modified from Maier, 1993a). **(A)** Medial view of parasagittal section; **(B)** horizontal section. These images show the nasal structure without facial exocranial (dermal) bones except for the maxilla and palatine bones. at, atrioturbinal; et I (pa), ethmoturbinal I pars anterior; et I (pp), ethmoturbinal I pars posterior; et II–IV, ethmoturbinals II–IV; etr, ethmoturbinal recess; ft, frontoturbinal; in, interturbinal; lh, lamina horizontalis; lsc, lamina semicircularis; mt, marginoturbinal; mx, maxillare; mxr, maxillary recess; mxt, maxilloturbinal; nac (pa), nasal capsule pars anterior; nac (pl), nasal capsule pars lateralis; nac I (pp), nasal capsule pars posterior; nph, nasopharyngeal duct; nt, nasoturbinal; pal, palatinum.

As for the general developmental pattern for mammals, initially, these turbinals and laminae appear as simple ridges along the lateral wall of the nasal capsule (Dieulafe, 1906). The nasal capsule, which is the rostral part of the chondrocranium, undergoes drastic morphological changes through ontogeny (Maier and Ruf, 2014; Van Valkenburgh et al., 2014; Smith et al., 2015). Morphogenesis of the nasal capsule in mammals is attributed to three mesenchymal condensations: the parietotectal cartilage aside from the tectum, paranasal cartilage, and orbitonasal lamina (De Beer, 1937; Reinbach, 1952b; Moore, 1981; Zeller, 1987; Rossie, 2006; Smith and Rossie, 2006, 2008; Van Valkenburgh et al., 2014). The nasal tectum of the parietotectal cartilage condenses in the rostrocaudal and mediolateral direction (De Beer, 1937; Smith and Rossie, 2008). As the mesenchyme condenses, the rostral ridge of the paranasal cartilages overlaps the parietotectal cartilage, and the caudal ridge of the paranasal cartilages overlaps the orbitonasal lamina (Smith and Rossie, 2008; Van Valkenburgh et al., 2014). As a result, the lamina semicircularis is formed rostrally and ethmoturbinal I is formed caudally within the nasal capsule. Subsequently, ethmoturbinals II to IV are formed rostrocaudally within the orbitonasal lamina (Rossie, 2006; Smith and Rossie, 2008; Van Valkenburgh et al., 2014). The nasal capsule then becomes gradually enclosed by exocranial facial bones (Maier and Ruf, 2014). Through prenatal ontogeny, the structure of each turbinal changes in shape and becomes complicated, filling the nasal cavity (Maier and Ruf, 2014). Prenatally, the nasal epithelium sinks at specific sites, where the initial folds are created. Within the initial folds, mesenchymal condensations constitute the primitive morphology of the chondral template of turbinals (Smith et al., 2020). Later, these mesenchymal condensations chondrify. In perinatal and postnatal stages, cartilages change their shape into lamellae (Smith et al., 2020). In the adult, the cartilaginous lamellae is fully ossified with the process of endochondral ossification except for the marginoturbinal and atrioturbinal (Voit, 1909; Martineau-Doizé et al., 1992; Ruf et al., 2015; Smith et al., 2020). Additional turbinals branch off from each turbinal, scroll, and fold and also merge with one another (Parker, 1885; Maier, 1980, 2000; Deleon and Smith, 2014; Maier and Ruf, 2014; Smith et al., 2020). The ossified remnant of the nasal capsule becomes the ethmoid bone (Patterson, 1977). An emerging consensus agrees with the bauplan (body plan) of cartilaginous nasal capsule having a tripartite composition: the anterior part (pars anterior), lateral part (pars lateralis), and posterior part (pars posterior) (Figure 1) (Maier, 1993a; Rossie, 2006; Smith and Rossie, 2006, 2008; Maier and Ruf, 2014; Van Valkenburgh et al., 2014).

The turbinate anatomy of various mammalian species at the adult stage has been described by the classic works of Paulli, providing a major source of current information on the diversity of mammalian turbinals (Paulli, 1900a,b, c). However, his studies erroneously interpreted the lamina semicircularis as a turbinal, due to the lack of observations on fetal stages of nasal structures (Maier and Ruf, 2014). Since the nasal structure becomes highly complicated, particularly during prenatal development, it is virtually impossible to correctly establish turbinate homologies between species solely by comparisons of adult anatomy (Maier and Ruf, 2014). In contrast, the turbinate structure in fetal stages is rather simple, and observations on fetal series allow us for tracing the structural changes of the nasal capsule and turbinals (Maier, 1993a; Macrini, 2014; Maier and Ruf, 2014). Thus, previous studies have emphasized the importance of comparative embryological approaches for understanding turbinate homology among mammals (Novacek, 1993; Maier, 1993a; Maier and Ruf, 2014). However, few studies incorporate fetal samples (Reinbach, 1952a, b; Maier, 1980; Smith and Rossie, 2008; Giannini et al., 2012; Maier and Ruf, 2014; Ruf et al., 2015; Ruf, 2020), possibly due to the difficulty in obtaining rare fetal samples.

Bats lack the prenatal information on turbinate anatomy with unresolved turbinate homology. They are the second most speciose order of mammals, exceeding 1,400 recognized species (Wilson and Mittermeier, 2019; Simmons and Cirranello, 2020). Phylogenetically, they are presently divided into two suborders, i.e., Yangochiroptera and Yinpterochiroptera (Springer et al., 2001; Teeling et al., 2002, 2003, 2005). Apart from most members of the family Pteropodidae of the Yinpterochiroptera, many bat species can use laryngeal echolocation. Most echolocating bat species emit their calls orally, but in some families, echolocation calls are emitted nasally (Brigham et al., 2004; Feldhamer et al., 2007). Olfactory capabilities in bats have been suggested to vary between species (Bhatnagar and Kallen, 1974a; Hutcheon et al., 2002). Bat turbinals have been studied by many authors (Grosser, 1900; Frick, 1954; Bhatnagar and Kallen, 1974a, b, 1975; Cooper and Bhatnagar, 1976; Göbbel, 2002; Giannini et al., 2006, 2012; Nelson et al., 2007; Smith et al., 2012; Eiting et al., 2014a; Curtis and Simmons, 2017; Curtis et al., 2020; Yohe et al., 2018), but the complex and diverse anatomy of bat turbinals has caused much confusion regarding their homology, possibly owing to the variations in echolocation behavior and olfactory functions (Curtis and Simmons, 2017; Curtis et al., 2020).

A handful of studies have attempted to discuss the homologies of bat turbinals (Bhatnagar and Kallen, 1974a; Kämper and Schmidt, 1977; Curtis and Simmons, 2017) using adult specimens; however, as noted earlier, homologies of the mammalian nasal structures are hardly possible to establish without studying fetal anatomy. To date, our knowledge on prenatal turbinals in bats is still in its infancy and restricted to only a few studies on some bat species, including *Rousettus aegyptiacus* (Jurgens, 1962; Fehse, 1990), *Pipistrellus pipistrellus*, *Rhinolophus ferrumequinum*, *Vespertilio murinus* (Grosser, 1900), *Miniopterus schreibersii* (Fawcett, 1919; De Beer, 1937), *Myotis myotis* (Frick, 1954), *Pteropus lylei* (Giannini et al., 2012), *Megaderma lyra* (Smith et al., 2012), and *Rousettus leschenaultii* (Smith et al., 2020).

Similarly, the fetal anatomy and ontogenetic periods to adult stages in bats are still largely unexplored or poorly studied. Here, using diffusible iodine-based contrast-enhanced computed tomography (diceCT) imaging, we describe the detailed embryonic development of the nasal cavity in eight species of bats, dividing into two suborders: Yangochiroptera and Yinpterochiroptera. We revise turbinate homologies among bats and reconstruct the evolutionary history of the nasal turbinal of bats in light of the modern phylogenetic framework.

## Materials and Methods

**FIGURE 2:**
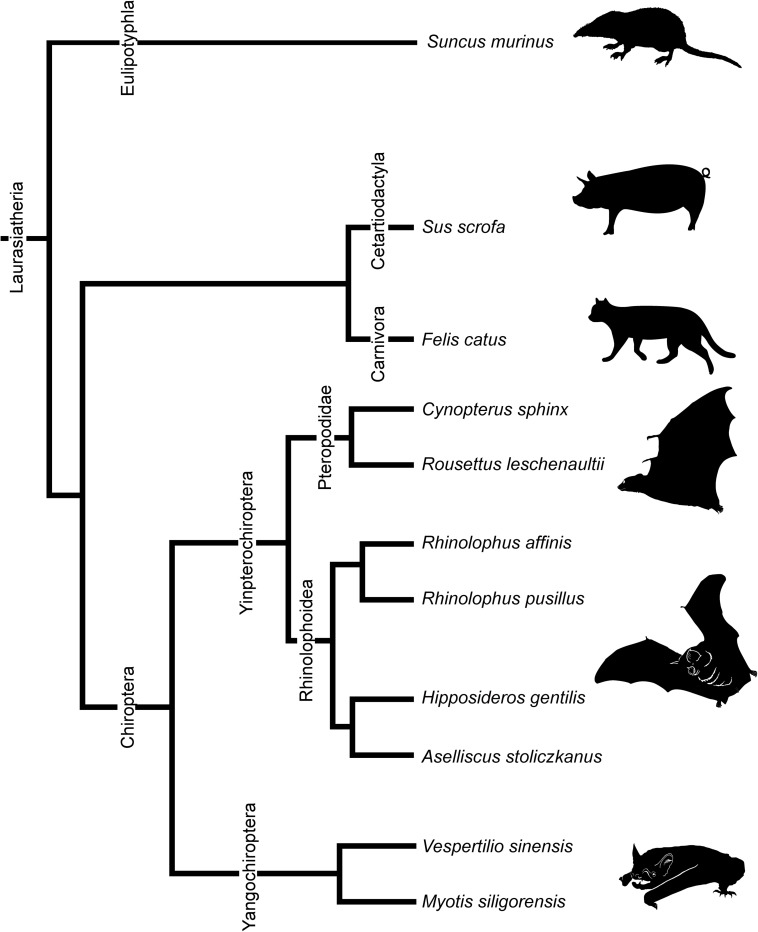
Phylogenetic relationships of bats and outgroup species in this study. Phylogenetic framework is based on Teeling et al. (2005) and Li et al. (2007).

We observed multiple developmental stages from the fetus to adult of eight species of bats. Stages and basic measurements are summarized in Supplementary Table 1. Our samples include *Cynopterus sphinx* and *R. leschenaultii* from Pteropodidae, *Rhinolophus affinis* and *Rhinolophus pusillus* from Rhinolophidae, *Hipposideros gentilis* and *Aselliscus stoliczkanus* from Hipposideridae of Yinpterochiroptera, and *Myotis siligorensis* and *Vespertilio sinensis* from Vespertilionidae species of Yangochiroptera (Figure 2). Fetuses of three non-bat species of Laurasiatheria, *Suncus murinus*, *Sus scrofa*, and *Felis catus*, were included as outgroups (Figure 2). Samples belong to the curatorial collections at the Institute of Ecology and Biological Resources of Vietnam Academy of Science and Technology and the University Museum of the University of Tokyo (Supplementary Table 1). These samples were fixed and preserved with 70% ethanol solution. Grayscale images of the specimens’ crania were obtained using microCT (InspeXio SMX-90CT Plus, Shimadzu Co, Japan) with 90 kV source voltage and 100 mA source currents. To enhance the contrast of the CT images, we followed the image enhancement techniques of a previous study (Gignac and Kley, 2014; Gignac et al., 2016) and dipped the specimens with iodine-based solutions (1% iodine, I_2_KI in 99% ethanol solution) (Sohn et al., in press). Staining duration was between 6 and 24 h depending on the size of the specimen. Voxel size ranged from 8 to 35 μm. Images were reconstructed with dimensions of 1,024 × 1,024 pixels and in 12-bit grayscale. We reconstructed the cartilage and bones within turbinals by manual segmentation of grayscale images for each specimen using Segmentation Editor Tool in Amira 5.3 (Visage Imaging, Berlin, Germany) (Supplementary Table 1). The cartilaginous structures are stained poorly by iodine-based solutions. We identified them indirectly from the connective tissue like perichondria, which are readily stained with iodine-based solutions (Gignac et al., 2016). When interpreted from the surrounding structure, it is possible to distinguish ossified and cartilaginous structures. Supplementary Figure 1 shows the ossified and cartilaginous structure with enhanced contrast of the CT images from iodine solution. The crown-rump length (CRL) of each specimen was measured using sliding calipers (N20, Mitutoyo, Japan). Bat specimens were staged following Cretekos et al. (2005), which has been developed based on the Carnegie system for human development. Bat fetal specimens of stages CS18, CS19, and CS22 or CS23 of Cretekos’ staging system (which respectively correspond to CS18, CS19, and CS22 in the human Carnegie system), in which turbinate development and splitting can be observed, were here compared. In this study, specimens assigned as stage 18 are hereafter referred to as “early stage,” stage 19 as “mid stage,” and stages 22 and 23 as “late stage” for simplification. For *R. pusillus*, a fetal specimen of stage 15 was additionally studied to observe the initial onset of the turbinate projection. Gestation day 29 and postnatal day 1 of *S. murinus* are respectively referred as “mid stage” and “late stage” (which roughly correspond to CS22 and CS23 in the human Carnegie system). Gestation day 28 and gestation day 40 of *S. scrofa* are respectively referred as “mid stage” and “late stage” (which roughly correspond to CS22 and CS23 in the human Carnegie system). Gestation day 38 and gestation day 49 of *F. catus* are referred as “mid stage” and “late stage,” respectively (which roughly correspond to CS22 and CS23 in the human Carnegie system). *S. scrofa* and *F. catus* were aged based on Evans and Sack (1973). Specimen ID, CRL, stages, and scanning parameters of all specimens are summarized in Supplementary Table 1.

### Terminology

The anatomical terminology for turbinals varies between studies (Table 1), but here we adopted the bauplan proposed by Maier (1993a) and followed the anatomical terminology of Voit (1909) (Figure 1). We chose this terminology because it takes into account the topography, ontogeny, and homology of turbinal bones (Maier and Ruf, 2014).

**TABLE 1:** Terminology for turbinals and laminas.

Structure name	Synonyms from other authors
Marginoturbinal	–
Atrioturbinal	–
Maxilloturbinal	Inferior concha (Moore, 1981, p. 255)
Nasoturbinal	Nasoturbinal, mucosal part (Smith and Rossie, 2008), rostral nasoturbinal (Giannini et al., 2012)
Lamina semicircularis	Crista semicircularis (Voit, 1909), endoturbinal I (Paulli, 1900a,b,c; Moore, 1981), semicircular crest (Smith and Rossie, 2008), caudal nasoturbinal (Giannini et al., 2012), nasoturbinal osseous part (Smith et al., 2015)
Lamina horizontalis	Anterior root of ethmoturbinal I (De Beer, 1937), lateral root of ethmoturbinal I (Rossie, 2006), frontomaxillary septum (Smith and Rossie, 2008), lamina transversalis posterior (Macrini, 2014)
Ethmoturbinal I pars anterior	Endoturbinal I (Allen, 1882; Giannini et al., 2012), endoturbinal II (Paulli, 1900a,b, c; Moore, 1981), middle concha (Moore, 1981, p. 255), ethmoturbinals I (Smith and Rossie, 2008), endoturbinal I in adult (Macrini, 2014)
Ethmoturbinal I pars posterior	Ethmoturbinal I lobule (Allen, 1882), endoturbinal II, lower lamella (Paulli, 1900a,b,c; Moore, 1981), middle concha (Moore, 1981, p. 255), ethmoturbinals II (Smith and Rossie, 2008), endoturbinal I in adult (Macrini, 2014)
Ethmoturbinal II	Endoturbinal II (Allen, 1882; Giannini et al., 2012), endoturbinal III (Allen, 1882; Paulli, 1900a,b,c; Moore, 1981), superior concha (Moore, 1981, p. 255), ethmoturbinals III (Smith and Rossie, 2008), endoturbinal II in adult (Macrini, 2014)
Ethmoturbinal III	Endoturbinal III (Allen, 1882; Giannini et al., 2012), endoturbinal IV (Paulli, 1900a,b,c; Moore, 1981), highest concha (Moore, 1981, p. 255), ethmoturbinal IV (Smith and Rossie, 2008)
Interturbinal	Ectoturbinal (Allen, 1882; Paulli, 1900a,b,c; Moore, 1981; Giannini et al., 2012)
Frontoturbinal	Ectoturbinal (Allen, 1882; Paulli, 1900a,b,c; Moore, 1981; Giannini et al., 2012), ectoturbinal in adult (Macrini, 2014)

## Results

### Marginoturbinal and Atrioturbinal

**FIGURE 3:**
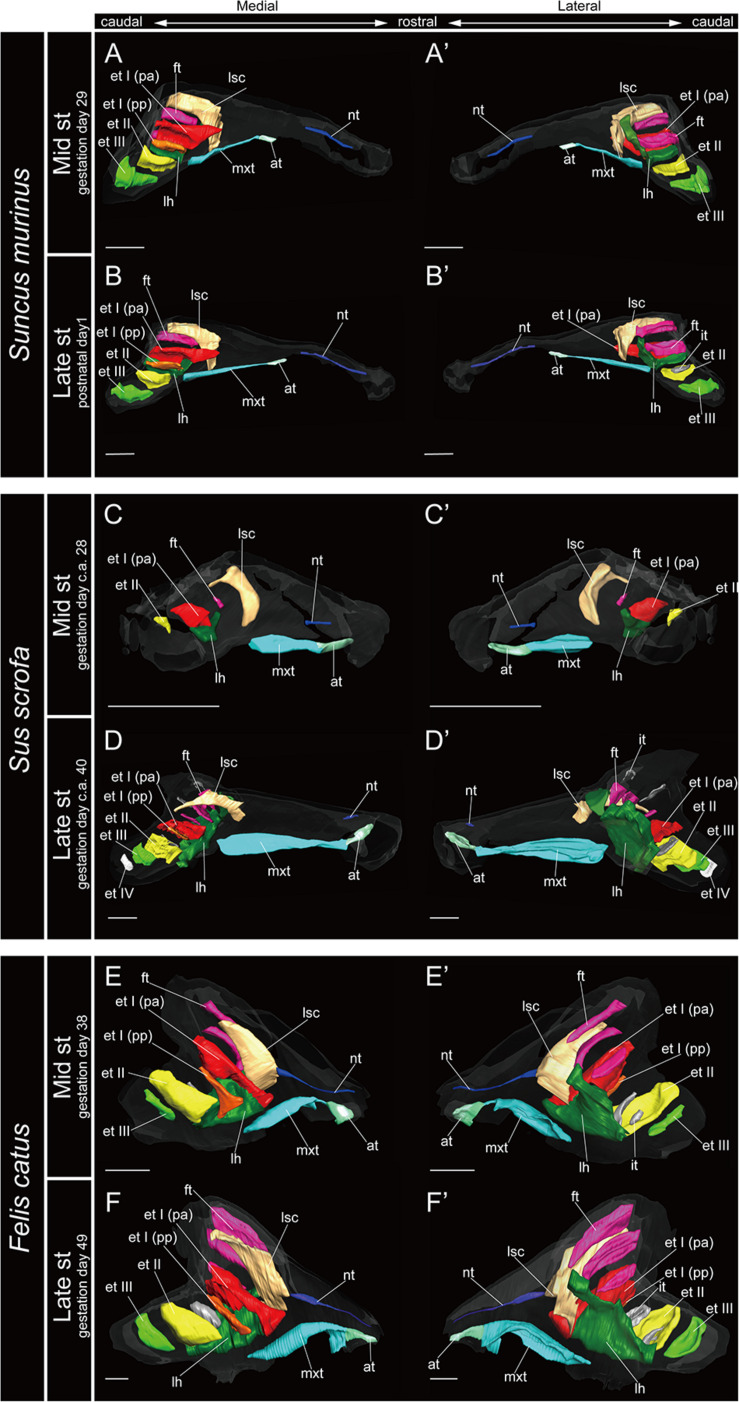
3D reconstructions of the developing turbinals in non-bat laurasiatherians. **(A,A′,C,C′,E,E′)** Mid stage fetus; **(B,B′,D,D′,F,F′)** late stage fetus or postnatal; **(A–F)** medial view; **(A′–F′)** lateral view. Scale bars, 1 mm. at, atrioturbinal; et I (pa), ethmoturbinal I pars anterior; et I (pp), ethmoturbinal I pars posterior; et II–IV, ethmoturbinals II–IV; ft, frontoturbinal; mxt, maxilloturbinal; lh, lamina horizontalis; nt, nasoturbinal; lsc, lamina semicircularis.

**FIGURE 4:**
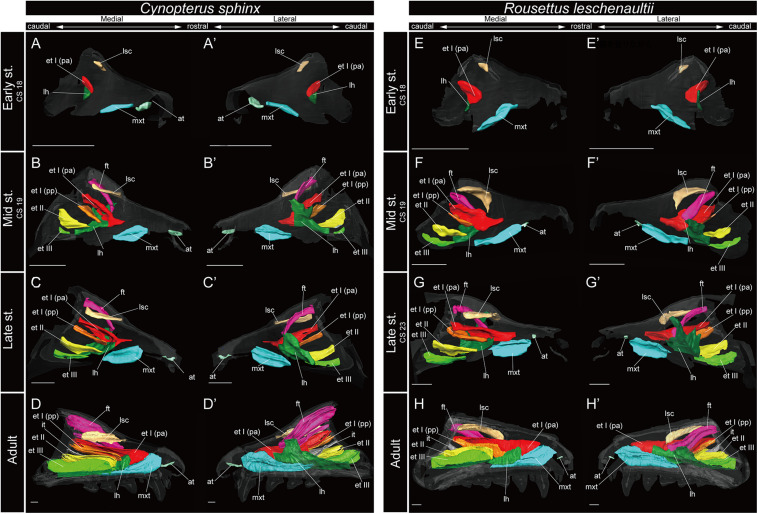
3D reconstructions of the developing turbinals in *Cynopterus sphinx* and *Rousettus leschenaultii*. **(A–D′)** *C. sphinx*; **(E–H′)** *R. leschenaultii*; **(A,A′,E,E′)** early stage fetus; **(B,B′,F,F′)** mid stage fetus; **(C,C′,G,G′)** late stage fetus; **(D,D′,H,H′)** adult. **(A–D)** Medial view and **(A′–D′)** lateral view of *C. sphinx*; **(E–H)** medial view and **(E′–H′)** lateral view of *R. leschenaultii*. Scale bars, 1 mm. at, atrioturbinal; et I (pa), ethmoturbinal I pars anterior; et I (pp), ethmoturbinal I pars posterior; et II–IV, ethmoturbinals II–IV; ft, frontoturbinal; mxt, maxilloturbinal; lh, lamina horizontalis; nt, nasoturbinal; lsc, lamina semicircularis.

**FIGURE 5:**
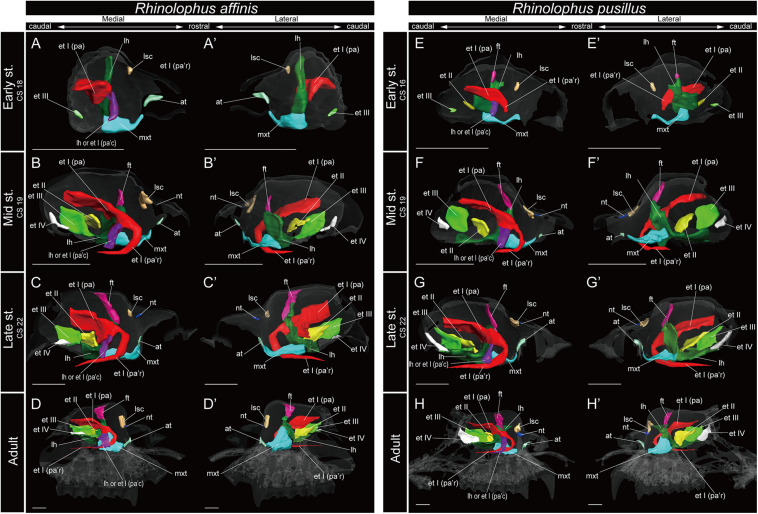
3D reconstructions of the developing turbinals in *Rhinolophus affinis* and *R. pusillus*. **(A–D′)** *R. affinis*; **(E–H′)** *R. pusillus*; **(A,A′,E,E′)** early stage fetus; **(B,B′,F,F′)** mid stage fetus; **(C,C′,G,G′)** late stage fetus; **(D,D′,H,H′)** adult. **(A–D)** Medial view and **(A′–D′)** lateral view of *R. affinis*; **(E–H)** medial view and **(E′–H′)** lateral view of *R. pusillus*. Scale bars, 1 mm. at, atrioturbinal; et I (pa), ethmoturbinal I pars anterior; et I (pa’c), caudal part of ethmoturbinal I pars anterior; et I (pa’r), rostral part of ethmoturbinal I pars anterior; et II–IV, ethmoturbinals II–IV; ft, frontoturbinal; mxt, maxilloturbinal; lh, lamina horizontalis; nt, nasoturbinal; lsc, lamina semicircularis.

**FIGURE 6:**
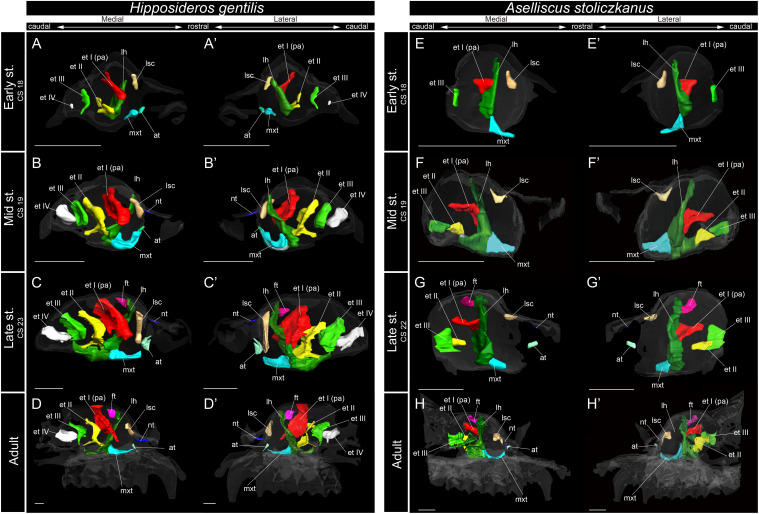
3D reconstructions of the developing turbinals in *Hipposideros gentilis* and *Aselliscus stoliczkanus*. **(A–D′)** *H. gentilis*; **(E–H′)** *A. stoliczkanus*; **(A,A′,E,E′)** early stage fetus; **(B,B′,F,F′)** mid stage fetus; **(C,C′,G,G′)** late stage fetus; **(D,D′,H,H′)** adult. **(A–D)** Medial view and **(A′–D′)** lateral view of *H. gentilis*; **(E–H)** medial view and **(E′–H′)** lateral view of *A. stoliczkanus*. Scale bars, 1 mm. at, atrioturbinal; et I (pa), ethmoturbinal I pars anterior; et I (pp), ethmoturbinal I pars posterior; et II–IV, ethmoturbinals II–IV; ft, frontoturbinal; mxt, maxilloturbinal; lh, lamina horizontalis; nt, nasoturbinal; lsc, lamina semicircularis.

**FIGURE 7:**
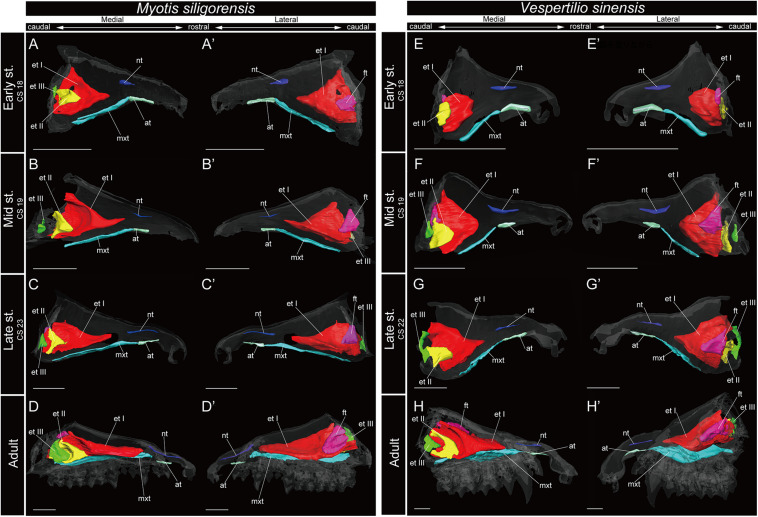
3D reconstructions of the developing turbinals in *Myotis siligorensis* and *Vespertilio sinensis*. **(A–D′)** *M. siligorensis*; **(E–H′)** *V. sinensis*; **(A,A′,E,E′)** early stage fetus; **(B,B′,F,F′)** mid stage fetus; **(C,C′,G,G′)** late stage fetus; **(D,D′,H,H′)** adult. **(A–D)** Medial view and **(A′–D′)** lateral view of *M*. *siligorensis*; **(E–H)** medial view and **(E′–H′)** lateral view of *V. sinensis*. Scale bars, 1 mm. at, atrioturbinal; et I (pa), ethmoturbinal I pars anterior; et I (pp), ethmoturbinal I pars posterior; et II–III, ethmoturbinal II–III; ft, frontoturbinal; mxt, maxilloturbinal; lh, lamina horizontalis; nt, nasoturbinal; lsc, lamina semicircularis.

The marginoturbinal and the atrioturbinal were cartilaginous structures in all species examined here. The atrioturbinal of all outgroup species and all bats was positioned ventrally, and it was continuous with the maxilloturbinal caudally (Figures 3–7). As the nasal capsule enlarged, the atrioturbinal became more rostrocaudally elongated in the outgroup species as well as in Pteropodidae (Figures 3, 4). The atrioturbinal is more rostrocaudally elongated from the early stage in *R. affinis* and *H. gentilis* and from the mid stage in *R. pusillus* (Figures 5A,A′,F,F′, 6A,A′). In addition, the atrioturbinal developed toward the rostrocaudal direction starting with the late stage (Figures 5, 6). While the atrioturbinal was visible, the marginoturbinal was partly visible in our scans. Hence, the marginoturbinal cannot be reconstructed. The contrast between the thick cartilage and surrounding soft tissue was not clear enough to identify the boundary.

### Maxilloturbinal

In most specimens examined in this study, the maxilloturbinal was positioned caudally to the atrioturbinal. The maxilloturbinal was a rostrally positioned structure within the nasal cavity, and its ventral side folded inward. The maxilloturbinal enlarged as it developed in all outgroup species (Figure 3 and Supplementary Figure 2). At the same time, it showed a double scroll in *S. scrofa* (Figures 3C–D′ and Supplementary Figures 2C,D) and a single scroll in *S. murinu*s and *F. catus* (Figures 3A–B′,E–F′ and Supplementary Figures 2A,B,E,F). The maxilloturbinal of the outgroup fetus was cartilaginous.

Among bats, the developmental pattern of Pteropodidae resembled that in outgroup species. The maxilloturbinal of Pteropodidae was the largest among all turbinals and laminae starting in the early stage (Figures 4A,A′,E,E′). Beginning at the mid stage in *C. sphinx* and late stage in *R. leschenaultii*, the maxilloturbinal started branching (Supplementary Figures 3B, 4C). From the mid stage in *C. sphinx* and late stage in *R. leschenaultii*, the maxilloturbinal developed dorsal and ventral branches which were both laterally scrolled as in the late stage of *S. scrofa* (Figures 3D,D′, 4B,B′,G,G′ and Supplementary Figures 2D, 3B, 4C). Also, the cartilaginous structure was replaced and ossified in the adult (Supplementary Figures 3, 4).

Within Rhinolophoidea, all species presented similar maxilloturbinal morphologies. The maxilloturbinal enlarged and only partially ossified in the early to late stages (Figures 5, 6 and Supplementary Figures 5–8). The maxilloturbinal also fused with the lamina horizontalis caudally such that it occurred in the early stage in *Rhinolopus* and *A. stoliczkanus* (Figures 5A,A′,E,E′, 6E,E′) and in the mid stage in *H. gentilis* (Figures 6B,B′). Nonetheless, the maxilloturbinal was reduced compared with other turbinals and laminae after the late stage in all species belonging to Rhinolophoidea (Figures 5, 6 and Supplementary Figures 5–9). In addition, only the caudal side of the maxilloturbinal was ossified in the adult, and the rostral side remained cartilaginous (Supplementary Figure 9).

The maxilloturbinal of *M. siligorensis* and *V. sinensis* was rostrocaudally elongated and lateromedially narrow (Figure 7). It was slim and rod-shaped from the early to late stages (Figures 7A–C′,E–G′). Unlike the outgroup and Pteropodidae, the maxilloturbinal did not branch, and it showed a single scroll ventrally as it developed from the late stage to adult (Supplementary Figures 2–4, 10, 11). It extended lateromedially, becoming a plate-like structure where it attached to the inner lateral nasal wall in the adult (Figures 7D,D′,H,H′ and Supplementary Figures 10, 11).

### Nasoturbinal

We observed the nasoturbinal in mid and late stages of the outgroup species. The nasoturbinal slightly projected ventrally from the nasal wall (Figure 3). It did not show any scrolling, and it extended rostrocaudally and was observed near the naris in both mid and late stages in all outgroups (Figure 3 and Supplementary Figure 2). The length of the nasoturbinal varied among the outgroup species such that the nasoturbinal of *S. murinus* and *F. catus* was rostrocaudally longer than *S. scrofa* (Figure 3). The nasoturbinal of *S. murinus* and *F. catus* elongated rostrocaudally such that its length was comparable to that of the maxilloturbinal (Figures 3A–B′,E–F′).

In Pteropodidae, the nasoturbinal was absent during prenatal developmental stages (Figure 4 and Supplementary Figures 3, 4). In the adult, a slight projection was observed dorsally to the nasal cavity near the naris (Supplementary Figures 3, 4).

The nasoturbinal of *Rhinolophus* and *H. gentilis* from the mid stage and of *A. stoliczkanus* from the late stage consisted of a tiny cartilaginous structure, continuing with the lamina semicircularis (Figures 5B–D′,F–H′, 6B–D′,G–H′ and Supplementary Figures 5–8).

The nasoturbinal of *M. siligorensis* and *V. sinensis* was much more well-developed than other chiropteran species and projected slightly ventrally (Figure 7). It did not show any scrolling and extended rostrocaudally beyond the atrioturbinal–maxilloturbinal contact. While it formed a short rod-like structure in the early and mid stages (Figures 7A–B′,E–F′ and Supplementary Figures 10, 11), in the late stage and adult, it formed a long rod-like structure rostrocaudally (Figures 7C–D′,G–H′ and Supplementary Figures 10, 11).

### Lamina Semicircularis

The lamina semicircularis was observed in all outgroup species (Figure 3). This lamina extended from the inner wall in the central region of the nasal capsule toward the lateromedial, dorsoventral, and caudorostral directions. It expanded transversally on the dorsal side of the nasal cavity as it developed from the mid stage to the late (Figure 3 and Supplementary Figure 2).

Among bats, the lamina semicircularis of *C. sphinx* and *R. leschenaultii* showed an almost identical developmental pattern as that of the outgroup species in terms of the transverse expansion. The lamina semicircularis was observed in the early stage for both species (Figures 4A,A′,E,E′ and Supplementary Figures 3, 4). The lamina semicircularis extended in the caudorostral direction rather than the dorsoventral direction which is different from the observation in the outgroup. While the lamina semicircularis of *C. sphinx* and *R. leschenaultii* was not as large as that of the outgroup species, it was as large as the frontoturbinal and the ethmoturbinal III from the mid stage (Figures 4B–D′,F–H′).

The developmental pattern of the lamina semicircularis was similar between Rhinolophoidea species (Figures 5, 6 and Supplementary Figures 5–8). The lamina semicircularis was observed in all fetal stages and in the adult of Rhinolophoidea (Figures 5, 6 and Supplementary Figures 5–8). The lamina semicircularis of *H. gentilis* was the largest among all Rhinolophoidea (Figures 5, 6 and Supplementary Figures 5–8). It projected ventrally from the inner wall of the nasal capsule starting in the early stage (Figures 6A,A′ and Supplementary Figure 7A). The lamina semicircularis then elongated toward the lateromedial and dorsoventral directions from the inner wall of the nasal capsule in the mid to late stages (Figures 6B–C′ and Supplementary Figures 7B,C). In the late stage, the lateromedially and dorsoventrally extended lamina semicircularis formed a wall that separated the anterior and the middle region of the nasal capsule (Figures 6C,C′ and Supplementary Figure 7C). In the adult, the lamina semicircularis was partly ossified, but not scrolled in all Rhinolophoidea (Figures 5, 6 and Supplementary Figures 5–8).

In the adult of *M. siligorensis* and *V. sinensis*, the lamina semicircularis was not observed in the caudal region of the nasal cavity, which was surrounded by the maxilla and palatine (Figures 7D,D′,H,H′ and Supplementary Figures 10, 11). The nasoturbinal was found in most of the dorsal region of the external nasal cartilage (rostral side of the nasal cavity), but the laminar structure was not seen in the caudal side of the nasoturbinal (Figures 7D,D′,H,H′ and Supplementary Figures 10, 11). While the nasoturbinal was observed, the laminar structure was also not observed in the caudal side of the nasoturbinal in any fetal stages (Figures 7A–C′,E–G′ and Supplementary Figures 10, 11). Hence, the lamina semicircularis was not formed in both *M. siligorensis* and *V. sinensis*.

### Lamina Horizontalis

The lamina horizontalis horizontally separates the nasopharyngeal duct and the ethmoturbinal recess, which includes several turbinals in the outgroup species (Figure 3 and Supplementary Figure 2). The lamina horizontalis of the outgroup species extended from the inner wall of the nasal capsule toward the lateromedial, rostrocaudal, and caudorostral directions. In the mid and late stages, the dorsal side of the lamina horizontalis fused with the ventral side of the ethmoturbinal I pars anterior and the ethmoturbinal I pars posterior except for the mid stage of *S. scrofa* (Figure 3 and Supplementary Figure 2).

Among bats, *C. sphinx* and *R. leschenaultii* showed a developmental pattern similar to the outgroup species, whereas the developmental patterns of Rhinolophoidea and Yangochiroptera were different from those of the outgroup species. Similar to the mid stage of *S. scrofa*, the lamina horizontalis projected from the inner wall of the nasal capsule in the early stage of *C. sphinx* and *R. leschenaultii*, and dorsally, it fused with the ventral side of the ethmoturbinal I pars anterior (Figures 3C,C′, 4A,A′,E,E′ and Supplementary Figures 2C, 3A, 4A). The lamina horizontalis likely extended toward the lateromedial, rostrocaudal, and caudorostral directions and fused with the ventromedial part of the ethmoturbinal I pars posterior and the ethmoturbinal II from the early to the mid stages (Figures 4A–B′,E–F′ and Supplementary Figures 3, 4). The lamina horizontalis enlarged lateromedially and elongated rostrocaudally from the mid stage to adult (Figures 4B–D′,F–H′ and Supplementary Figures 3, 4). In all fetal stages, it was cartilaginous in both species, but ossified in the adult (Supplementary Figures 3, 4).

The lamina horizontalis of Rhinolophoidea showed a different developmental pattern compared with that of all outgroups and Pteropodidae (Figures 3–6). In all members of Rhinolophoidea, the lamina horizontalis that projected from the inner wall of the nasal capsule extended toward the lateromedial and dorsoventral directions from the early stage to adult. The lamina horizontalis formed a wall perpendicular to the rostrocaudal plane (Figures 5, 6 and Supplementary Figures 5–8).

Among Rhinolophoidea, the presumptive developmental pattern of the lamina horizontalis of *Rhinolophus* was somewhat dissimilar to that of *H. gentilis* or *A. stoliczkanus*, such that the lamina horizontalis scrolled ventrolaterally beginning in the early stage (Figures 5A,A′,E,E′ and Supplementary Figures 5A, 6A). The apex of the lamina horizontalis projected inward in the early stage and then gradually extended ventrally and medially from the mid stage to the adult (Figure 5 and Supplementary Figures 5, 6). In the adult, its apex extended caudally into the nasopharyngeal duct (Supplementary Figures 5, 6). When observed medially from the sagittal plane, it formed a hairpin-shaped structure (Figures 5D,D′,H,H′). When seen in coronal sections, the lamina horizontalis extended from the dorsolateral side of the nasal wall toward the ventromedial side. At the medial portion of the nasal capsule, the ethmoturbinal I pars anterior is positioned dorsally from the lamina horizontalis from the early stage of *Rhinolophus* (Supplementary Figures 5, 6). The ventromedial part of the lamina horizontalis bent lateralward from the point in which the ethmoturbinal I pars anterior extended from the late stage to adult in *R. affinis* and from mid sage to adult of *R. pusillus* (Supplementary Figures 5, 6). At the caudal portion of the nasal cavity of adult *Rhinolophus*, the ventral edge of the ventromedial part of the lamina horizontalis was round and appeared inside of the nasopharyngeal duct (Supplementary Figures 5, 6). Nonetheless, this hairpin-shaped structure of the lamina horizontalis may be the ethmoturbinal I pars anterior (Figure 5). The structure which is most probably the lamina horizontalis was ossified in the adult (Supplementary Figures 4D, 5D, 9).

The lamina horizontalis was not observed in *M. siligorensis* and *V. sinensis* in any fetal stages or the adult, unlike all outgroup species as well as Pteropodidae and Rhinolophoidea (Figure 7 and Supplementary Figures 10, 11).

### Ethmoturbinal I Pars Anterior

The ethmoturbinal I pars anterior of the outgroup species projected toward the lateromedial and caudorostral directions from the inner wall of the nasal capsule already in the mid stage, fusing with the dorsal side of the lamina horizontalis (Figure 3 and Supplementary Figure 2). It was the largest turbinal among all ethmoturbinals from the mid to late stages in all outgroups (Figure 3). The ethmoturbinal I pars anterior of *S. scrofa* was less developed than that of *F. catus* and *S. murinus* in the mid stage (Figures 3A,A′,C,C′,E,E′).

The ethmoturbinal I pars anterior of Pteropodidae showed the same developmental pattern as that of the outgroup species (Figures 3, 4). The developmental pattern from the early to mid stages, in particular, resembled that from the mid to the late stages of *S. scrofa* (Figures 3C–D′, 4A–B′,E–F′). The ethmoturbinal I pars anterior of Pteropodidae fused ventrally with the lamina horizontalis and projected toward the lateromedial direction from the inner wall of the nasal capsule (Figures 4A–B′,E–F′). The ethmoturbinal I pars anterior extended rostrally from the early to mid stages, and in the adult, it reached as far as the dorsal border of the maxilloturbinal (Figures 4A–B′,D,D′,E–F′,H,H′). The ethmoturbinal I pars anterior of Pteropodidae was the largest turbinal among all ethmoturbinals from the early stage to the adult (Figure 4). The ethmoturbinal I pars anterior was ossified in the adult (Supplementary Figures 4, 5).

**FIGURE 8:**
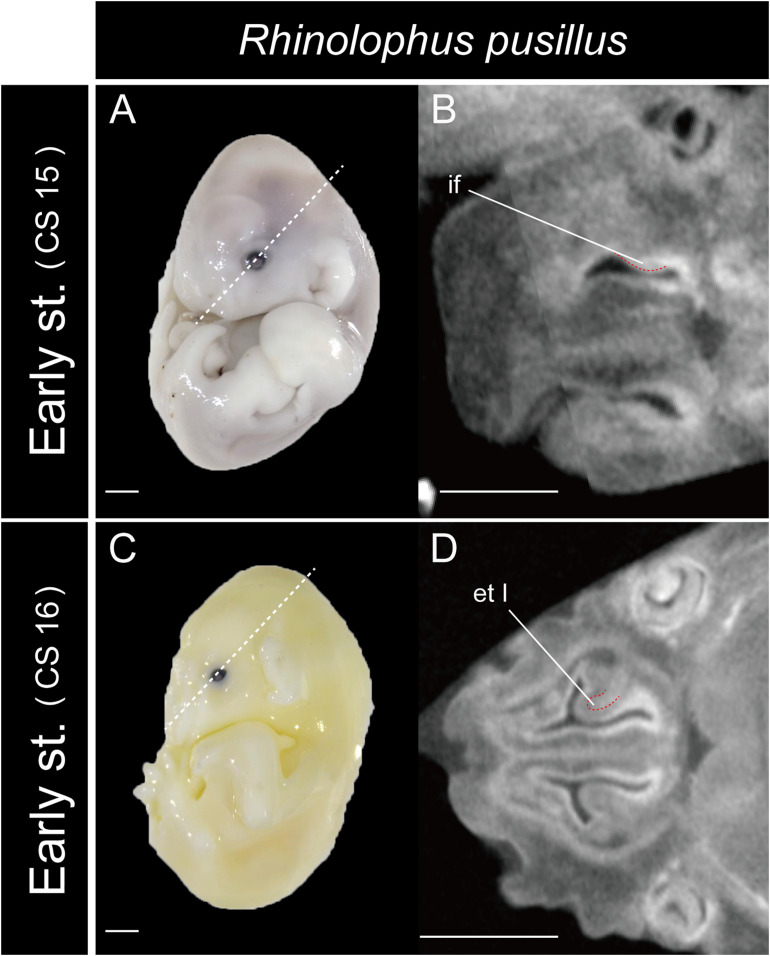
The onset of the turbinate projections in *Rhinolophus pusillus*. Broken lines indicate the location of section. Scale bar, 1 mm. **(A)** Early stage (CS 15) fetus embryo; **(B)** early stage fetus (CS 15) cross section; **(C)** early stage fetus (CS 16) embryo; **(D)** early stage fetus (CS 16) cross section. et I, ethmoturbinal I; if, initial fold.

In *Rhinolophus*, the developmental pattern of the ethmoturbinal I pars anterior differed from that of all other species and formed a distinctive structure (Figure 5). The early stage (CS 15) of *R. pusillus* was the smallest of all samples examined in this study (Figures 8A,B). In the early stage (CS 15) of *R. pusillus*, the cartilage of the nasal capsule was obscured in the scan, but an initial fold was observed in the inner wall of the nasal capsule (Figure 8B). The cartilage which we believe as the ethmoturbinal I pars anterior of the late phase of early stage (CS 16) was likely embedding within this initial fold of the early stage (CS 15) in *R. pusillus* (Figures 8C,D).

The fusion of the ethmoturbinal I pars anterior with the lamina horizontalis extended toward the lateromedial direction from the inner wall in the early stage of *Rhinolophus*, like the mid stage of *S. scrofa*, and the early stage of Pteropodidae (Figures 3–5, and Supplementary Figures 2–6). The alternative interpretation is that the lamina horizontalis did not form the hairpin-shaped structure in medial side. Consequently, the ethmoturbinal I pars anterior split into rostral and caudal parts from the early stage (Figures 5A,A′,E,E′ and Supplementary Figures 5, 6). In this case, the rostral part of the ethmoturbinal I pars anterior extended rostrally and curved ventrally in the mid stage of *R. affinis* and the early stage of *R. pusillus* (Figures 5B,B′,E,E′). Subsequently, it entered the nasopharyngeal duct, extending caudally in the mid stage of *R. affinis* and the late stage of *R. pusillus* (Supplementary Figures 5B, 6C). When observed medially from the sagittal plane, it formed a hairpin-shaped structure (Figures 5B,B′,E,E′). The apex of the rostral part of the ethmoturbinal I pars anterior extended caudally also from the mid stage (Figures 5B,B′,E,E′). It formed the freestanding structure in the adult (Figure 5).

The caudal part of the ethmoturbinal I pars anterior, which can be identified as a part of the lamina horizontalis in the alternative interpretation, scrolled medially in the mid stage of *R. affinis* and *R. pusillus* (Figures 5B,B′,F,F′ and Supplementary Figures 5, 6). Subsequently, the apex of the caudal part of the ethmoturbinal I pars anterior that projected inward gradually extended ventrally and medially until the late stage (Figure 5). In the adult, this apex extended caudally and became another freestanding structure in the nasopharyngeal duct (Figures 5D,D′,H,H′ and Supplementary Figures 5, 6). From the sagittal plane, it also formed a hairpin-shaped structure medially (Figures 5D,D′,H,H′). When seen in coronal sections, ethmoturbinal I pars anterior was present at the rostral portion of the nasal capsule. At the medial portion of the nasal capsule, the rostral and caudal part of ethmoturbinal I pars anterior was branched off from the lamina horizontalis. The rostral part extended toward the dorsomedial side, and the caudal part toward the ventromedial side from the early stage to adult (Supplementary Figures 5, 6). At the caudal portion of the nasal cavity of adult, the ventral edge of the caudal part of ethmoturbinal I pars anterior was round and appeared inside of the nasopharyngeal duct medially to the rostral part of ethmoturbinal I pars anterior (Supplementary Figures 5, 6).

In the early stage of *H. gentilis* and *A. stoliczkanus*, the ventral side of the ethmoturbinal I pars anterior fused at the dorsal side of the dorsoventrally elongated lamina horizontalis and projected from the inner wall of the nasal capsule (Figures 6A,A′,E,E′ and Supplementary Figures 7, 8). The ethmoturbinal I pars anterior was large after the early stage, and it developed dorsally and caudally to the lamina horizontalis in *H. gentilis* and *A. stoliczkanus*, respectively (Figures 6A,A′,E,E′). Still, it did not elongate rostrally like that of *S. murinus* and Pteropodidae (Figures 3A–B′, 4, 5). The ethmoturbinal I of Rhinolophoidea was ossified in the adult (Supplementary Figures 5–8).

Similar to the outgroup species and other bats, the ethmoturbinal I of *M. siligorensis* and *V. sinensis* was the largest turbinal from the early stage to adult (Figure 7). On the other hand, it differed from the outgroup species and Pteropodidae in that it did not split into pars anterior and pars posterior (Figures 3, 4, 7 and Supplementary Figures 2–4, 10, 11). The ventral side of the rostral end of ethmoturbinal I formed a horizontal plate-like process from the mid stage to adult (Figures 7B–D′,F–H′ and Supplementary Figures 10, 11). In addition, since the lamina horizontalis was absent in all stages, ethmoturbinal I projected solely from the inner wall of the nasal capsule (Figure 7 and Supplementary Figures 10, 11). While it enlarged dorsoventrally from the early to late stages (Figures 7A–C′,E–G′ and Supplementary Figures 10, 11), ethmoturbinal I extended toward the caudorostral direction from the late stage to adult (Figures 7C–D′,G–H′ and Supplementary Figures 10, 11). Ethmoturbinal I of Yangochiroptera was ossified in the adult (Supplementary Figures 10, 11).

### Ethmoturbinal I Pars Posterior

The ethmoturbinal I pars posterior was observed from the ventral side of the ethmoturbinal I pars anterior starting in the mid stage of *S. murinus* and *F. catus* and in the late stage of *S. scrofa* (Figures 3A,A′,D,D′,E,E′ and Supplementary Figures 2A,D,E).

Ethmoturbinal I pars posterior of Pteropodidae was located at the same place as that of the outgroup species (Figures 3, 4). It was absent in the early stage (Figures 4A,A′,E,E′ and Supplementary Figures 3, 4) but appeared starting in the mid stage (Figures 4B,B′,F,F′ and Supplementary Figures 3, 4).

In contrast to the outgroup species and Pteropodidae, ethmoturbinal I pars posterior was absent from the early stage to adult in all members of Rhinolophoidea, *M. siligorensis*, and *V. sinensis* (Figures 5–7).

### Turbinals in the Ethmoturbinal Recess

Ethmoturbinals II and III projected from the inner wall of the nasal capsule starting in the mid stage in *S. murinus* and *F. catus* (Figures 3A,A′,E,E′ and Supplementary Figures 2A,E). In *S. scrofa*, ethmoturbinal II was observed in the mid stage, while ethmoturbinal III was only present in the late stage (Figures 3C–D′ and Supplementary Figures 2C,D). In the outgroup species, ethmoturbinal II was the second largest among all ethmoturbinals after ethmoturbinal I pars anterior except for *S. murinus* (Figure 3).

In Pteropodidae, ethmoturbinal II and ethmoturbinal III were absent in the early stage; however, they projected from the inner wall of the nasal capsule after the mid stage (Figure 4 and Supplementary Figures 3, 4). In terms of size, ethmoturbinal II was larger compared with ethmoturbinal III in Pteropodidae in the mid and late stages (Figures 4B–C′,F–G′). Ethmoturbinal III was larger than ethmoturbinal I pars posterior from the late stage to adult (Figures 4C–D′,G–H′).

In *R. affinis*, ethmoturbinal II appeared from the inner wall of the nasal capsule in the mid stage, while in *R. pusillus*, it was observed in a similar position in the early stage (Figures 5B,B′,E,E′ and Supplementary Figures 5B, 6A). In both species, ethmoturbinal III projected from the inner wall of the nasal capsule in the early stage (Figures 5A,A′,E,E′ and Supplementary Figures 5, 6). In *R. pusillus*, the size of ethmoturbinal II and ethmoturbinal III was mostly comparable in the early stage (Figures 5E,E′). Ethmoturbinal III was larger compared with ethmoturbinal II in the mid stage (Figures 5B,B′,F,F′). Also, ethmoturbinal IV arose in the mid stage of *Rhinolophus* (Figures 5B,B′,F,F′ and Supplementary Figures 5, 6). Ethmoturbinal III was the largest, and ethmoturbinals II and IV were of the same size among these three turbinals from the late stage *Rhinolophus* (Figures 5C,C′,G,G′).

In *H. gentilis*, ethmoturbinals II, III, and IV projected from the inner wall of the nasal capsule in the early stage (Figures 6A,A′). Ethmoturbinal III was large in the early stage (Figures 6A,A′). Moreover, in *H. gentilis*, ethmoturbinal II was larger than ethmoturbinal III in the mid stage; however, the size became similar from the late stage (Figures 6B–D′). In *A. stoliczkanus*, ethmoturbinal III appeared in the early stage (Figures 6E,E′). Then, in the mid stage, ethmoturbinal II appeared rostrally to ethmoturbinal III (Figures 6F,F′). Ethmoturbinal IV was not observed from the early stage to adult (Figures 6H,H′).

Ethmoturbinal II appeared from the inner wall of the nasal capsule in the early stage of *M. siligorensis* and *V. sinensis*; however, after the mid stage, ethmoturbinal II shifted laterally, approaching ethmoturbinal I during late ontogeny as the nasal capsule enlarged (Figures 7B–D′,F–H′ and Supplementary Figures 10, 11). The caudal part of ethmoturbinal II curved and fused with the laterally positioned ethmoturbinal I from the late stage to adult (Figures 7C–D′,G–H′ and Supplementary Figures 10, 11). Both ethmoturbinal II and ethmoturbinal III extended toward the caudorostral and the ventrodorsal directions, forming a triangular shape from the mid stage (Figures 7B–D′,F–H′).

## Discussion

### Turbinate Ontogeny and Homology in Laurasiatheria and Pteropodidae

We found that the turbinate structures are principally comparable between Laurasiatheria and Pteropodidae. The fetuses of outgroup species, *S. murinus*, *S. scrofa*, and *F. catus*, together with the fetuses of Pteropodidae, appear to share a ventrally positioned and enlarged maxilloturbinal, which is the largest among all turbinals (Figures 3, 4 and Supplementary Figures 2–4). Ethmoturbinal I is the largest to develop among ethmoturbinals, splitting into pars anterior and pars posterior in all outgroup species (Figures 3A–B′,D–F′). In our study, adult specimens of outgroup species were not examined. Previous studies showed the ventrally positioned, branched off, and enlarged maxilloturbinal and ethmoturbinal I pars anterior and pars posterior in the adult *S. murinus* (Kuramoto, 1980), *S. scrofa* (Paulli, 1900a), and *F. catus* (Moore, 1981). These characteristics were also seen in the adult Pteropodidae studied here. Furthermore, the lamina horizontalis of the outgroup species and Pteropodidae divides the nasopharyngeal duct and the ethmoturbinal recess that includes several turbinals (Figures 3, 4 and Supplementary Figures 2–4). The enlarged ethmoturbinal recess with a vast space formed dorsally which is filled with the olfactory mucosa suggests that Pteropodidae are equipped with high olfactory ability (Negus, 1958; Craven et al., 2007, 2010; Eiting et al., 2014b). The developmental structure and position of the turbinal and lamina of the outgroup species studied here are congruent with the generality seen among previously reported non-volant mammals (Paulli, 1900a,b,c; Voit, 1909; Moore, 1981; Smith and Rossie, 2008; Van Valkenburgh et al., 2014).

Some authors mention the possibility that the unique turbinate morphology of *Rhinolophus* (which was also seen in our study) is related to echolocation (Curtis and Simmons, 2017; Curtis et al., 2020). Given this, and the fact that *Rhinolophus* belongs to Yinpterochiroptera as do Pteropodidae, we expected to see similar morphology in echolocating *Rousettus* prior to our experiment (Feldhamer et al., 2007). However, such behavioral differences (*Rousettus* is known for using tongue clicks for echolocation, while *Cynopterus* does not engage in such behavior) are not reflected in the turbinate structures of *R. leschenaultii* and *C. sphinx* (Figure 4 and Supplementary Figures 3, 4).

Based on adult specimens, Allen (1882) and Paulli (1900c) suggested that Pteropodidae [*Cynopterus*, *Epomophorus gambianus*, *Pteropus giganteus*, *Pteropus* sp., *Rousettus* (*Cyonycteris*)] have “four endoturbinals.” Given Allen’s (1882) schematic for the turbinal of *E. gambianus*, we assume that he incorrectly split the true ethmoturbinal I into “endoturbinal I” and “endoturbinal II” (Supplementary Figure 12). Allen (1882) drew other schematics for the turbinal of non-volant mammals, in which the author identified ethmoturbinal I pars anterior and pars posterior as the “endoturbinal I and lobule.” This suggests that the misidentification of the turbinal of *E. gambianus* reported by Allen (1882) was clearly not caused by the difference in nomenclature. Only tentative inferences can be made, as Paulli (1900c) provided no schematic for the turbinals of Pteropodidae, but we assume Paulli labeled the lamina semicircularis as “endoturbinal I.” Thus, there are “four endoturbinals” for the Pteropodidae species in Paulli’s view. Giannini et al. (2012) identified the turbinals based on a histological section of one fetal stage of *Pteropus* sp. and a CT-scanned image of the adult *P. lylei*. They pointed out that there is one additional “endoturbinal” in both studies of Allen (1882) and Paulli (1900c) compared with their observation (Figures 4C–D′,G–H′ and Supplementary Figure 12). Our observation on the turbinals of *C. sphinx* and *R. leschenaultii* shows that ethmoturbinal III projects from the inner nasal wall from the mid stage (Figures 4B–D′,F–H′ and Supplementary Figures 3, 4). Following this, no more ethmoturbinals are formed (Figure 4 and Supplementary Figures 3, 4). Allen’s study on *E. gambianus* (with our assumption that ethmoturbinal I splits into two parts; Supplementary Figure 12), Giannini’s study on *Pteropus*, and our study on *C. sphinx* and *R. leschenaultii* indicate that the number of ethmoturbinals is three in Pteropodidae (Figure 4 and Supplementary Figures 3, 4).

Giannini et al. (2012) compared *Pteropus* with non-bat mammals and claimed that the turbinate element composition in *Pteropus* is comparable with that of non-human primates (Smith and Rossie, 2006) and rodents (Paulli, 1900c) in terms of the reduced number of turbinals. They also concluded that the number of frontoturbinals in *Pteropus* differs from that of the hedgehog *Erinaceus* (Paulli, 1900c) and the marsupial *Monodelphis* (Rowe et al., 2005; Macrini, 2014) such that *Pteropus* has one while the hedgehog and marsupial have two frontoturbinals. The turbinate structure of *Pteropus* is both similar and different from that of carnivorans and ungulates which have increasing number and complexity (Stößel et al., 2010) in ethmoturbinals, frontoturbinals, and interturbinals (Giannini et al., 2012). Carnivorans have three ethmoturbinals like Pteropodidae, which are similar to our results (Paulli, 1900a,b,c; Wagner and Ruf, 2019, 2020) (Supplementary Table 1). However, Paulli reported that the maximum of the frontoturbinal and interturbinal combined (ectoturbinal in Paulli) is five to ten (only *Meles*) (Paulli, 1900c). This is different to Pteropodidae with one frontoturbinal and one interturbinal. The number of ethmoturbinal of ungulates varies such that *Capra* has three and *Dicotyles labiatus* has seven. Moreover, the number of frontoturbinal and interturbinal largely varies with seven in *Tragulus javanicus* and 31 in *Equus caballus* (Paulli, 1900c; Moore, 1981). *Capra* showed the same number of ethmoturbinal as Pteropodidae; however, the number of ethmoturbinal and frontoturbinal and interturbinal combined in ungulates is generally larger than that of Pteropodidae. Nonetheless, Giannini et al. (2012) pointed out that the turbinate homology of elements in *Pteropus* can be traced without difficulty based on the position of turbinals in canids (*Vulpes vulpes*). Even though the number varies for certain turbinals (caudal ethmoturbinal, interturbinal, and frontoturbinal) among non-volant Laurasiatheria and Pteropodidae, the turbinal element composition of pteropodids is easily traceable from that of our non-volant laurasiatherians. Therefore, we agree with Giannini et al. (2012) that the turbinate element composition of Pteropodidae is rather similar to that of other mammals (even for species with complex turbinals).

### Yangochiroptera

The lamina horizontalis is not formed throughout ontogeny in the studied Vespertilionidae (Figure 7 and Supplementary Figures 10, 11). Consequently, there is no clear separation between the nasopharyngeal duct and the ethmoturbinal recess. This anatomical setting suggests that these species are not specialized to keep the inspired air within the nasal cavity, allowing for better odorant sorption (Negus, 1958; Adams, 1972; Craven et al., 2010; Eiting et al., 2014b). Based on the nasal cavity structure, we suggest that the studied Vespertilionidae members are less capable of catching odorants compared with the outgroup mammals and Pteropodidae which have the lamina horizontalis and an independent space of the ethmoturbinal recess.

Fetal turbinals of *P. pipistrellus*, *V. murinus* (Grosser, 1900), *M. schreibersii* (Fawcett, 1919; De Beer, 1937), and *M. myotis* (Frick, 1954) were studied previously using histological sections. The figures given by Fawcett (1919); De Beer (1937), and Frick (1954) did not show the lamina, which separates the nasopharyngeal duct and the ethmoturbinal recess that includes several turbinals in these Vespertilionidae species. The lamina horizontalis separates the nasopharyngeal duct and the ethmoturbinal recess in all stages in the outgroup species and after mid stage in Pteropodidae (Figures 3, 4 and Supplementary Figures 2–4). Our study is consistent with the observation of Fawcett (1919); De Beer (1937), and Frick (1954). In the early stage of *M. siligorensis* and *V. sinensis*, ethmoturbinal II is located on the inner wall of the nasal capsule (Figures 7A,A′,E,E′ and Supplementary Figures 10, 11), but the caudal part of ethmoturbinal II curves and fuses with the laterally positioned ethmoturbinal I from the late stage to adult (Figures 7C–D′,G–H′ and Supplementary Figures 10, 11). As identified by Fawcett (1919) and De Beer (1937) in *M. schreibersii* and Frick (1954) in *M. myotis*, our study confirms that ethmoturbinal II is positioned medially to ethmoturbinal I in all fetal stages and adult in *M. siligorensis* and *V. sinensis.*

The present study did not cover members of Phyllostomidae, Emballonuridae, Molossidae, and Nycteridae, which also belong to Yangochiroptera (Teeling et al., 2002, 2003, 2005). Although most bats of Yangochiroptera emit sonar from their oral apparatus, Phyllostomidae and Nycteridae are characterized by emitting sonar from the naris (Jones and Teeling, 2006; Feng et al., 2012). As for the turbinals of Nycteridae, Allen (1882) is the only study that identifies turbinals in this family, in which the author described the adult *Nycteris thebaica*. He stated that *N. thebaica* has two endoturbinals (endoturbinal I has a lobule) and one ectoturbinal. Moreover, he claimed that it has a nasoturbinal that is larger than the endoturbinal. Regarding Phyllostomidae, Bhatnagar and Kallen (1974a) studied *Artibeus jamaicensis*; Kämper and Schmidt (1977) studied *Artibeus lituratus*, *Carollia perspicillata*, *Glossophaga soricina*, *Phyllostomus discolor*, and *Sturnira lilium*; and Yohe et al. (2018) studied *A. jamaicensis*, *Brachyphylla pumila*, *Erophylla bombifrons*, and *Phyllonycteris poeyi* of Phyllostomidae. Principally, the members of Phyllostomidae reportedly have seven turbinals. Although details of the identification varies among studies, all these studies agree that in Phyllostomidae the ethmoturbinal recess within the nasal cavity is separated with the nasopharyngeal duct rostrally by the ethmoturbinal and caudally by the lamina (Bhatnagar and Kallen, 1974a; Kämper and Schmidt, 1977; Yohe et al., 2018). Presenting coronal sections of the ethmoturbinal recess in three bats (*Anoura geoffroyi*, *S. lilium*, *Uroderma bilobatum*), Eiting et al. (2014a) showed that the lamina that separates the nasopharyngeal duct and the ethmoturbinal recess is well-developed. However, these studies are all based on adult species, and fetal information of Phyllostomidae is still largely lacking (Bhatnagar and Kallen, 1974a; Kämper and Schmidt, 1977; Eiting et al., 2014a; Yohe et al., 2018).

Compared with the patterns reported for Phyllostomidae, we recognize that *M. siligorensis* and *V. sinensis* do not have the lamina horizontalis, which is the lamina separating the nasopharyngeal duct and ethmoturbinal recess (Figure 7). This was particularly obvious in the caudal region of the nasal cavity (Bhatnagar and Kallen, 1974a; Kämper and Schmidt, 1977; Eiting et al., 2014a; Yohe et al., 2018). Further observation of the nasal development of Phyllostomidae, Mollossidae, Emballonuridae, and Nycteridae is required to clarify the whole picture of turbinate homology within Yangochiroptera.

### Rhinolophoidea

The rostral part of the lamina horizontalis extends dorsally in Rhinolophoidea from the early stage (Figures 5, 6A–A′,E–E′ and Supplementary Figures 5–8). The lamina horizontalis pushes the ethmoturbinal recess back toward the caudal direction, resulting in a small ethmoturbinal recess. As the size of the ethmoturbinal recess likely relates to olfactory ability (Negus, 1958; Adams, 1972; Craven et al., 2010; Eiting et al., 2014b), it is likely that Rhinolophoidea may have a reduced olfactory ability compared with the outgroup species and Pteropodidae.

**FIGURE 9:**
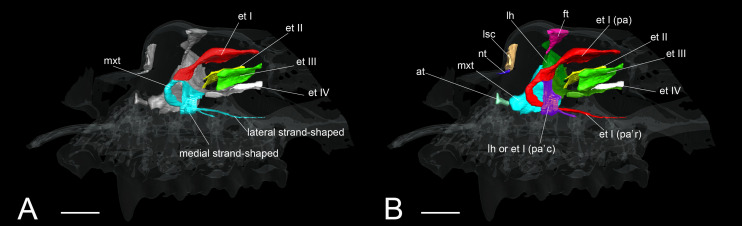
The identification of turbinals and laminas in *Rhinolophus affinis* by Curtis and Simmons (2017) and this study. **(A)** Descriptions by Curtis and Simmons (2017) with strand-shaped maxilloturinal; **(B)** revised descriptions by this study. Scale bar, 1 mm. et I (pa), ethmoturbinal I pars anterior; et I (pa’c), caudal part of ethmoturbinal I pars anterior; et I (pa’r), rostral part of ethmoturbinal I pars anterior; et II–IV, ethmoturbinals II–IV; ft, frontoturbinal; mxt, maxilloturbinal; lh, lamina horizontalis; lsc, lamina semicircularis.

Rhinolophoidea are undoubtedly the most disputed and problematic taxon among bats regarding its turbinate homology. Members of the superfamily Rhinolophoidea, which include Rhinolophidae, Hipposideridae, Megadermatidae, Craseonycteridae, and Rhinopomatidae, emit echolocation pulses from the naris (Csorba et al., 2003; Teeling et al., 2005; Li et al., 2007). The relationship between this behavior and their turbinal anatomy has not been understood (Curtis and Simmons, 2017; Curtis et al., 2020). Until today, very few have studied the turbinals of Rhinolophoidea, and there is some confusion in the literature regarding their turbinal homology. Studying *R. ferrumequinum* and *Rhinolophus hipposideros*, Grosser (1900) described the maxilloturbinal as a freestanding structure within the nasopharyngeal duct (Supplementary Figure 13). Following Grosser’s identification, recent studies on Rhinolophidae identified the freestanding structure in the nasopharyngeal duct as the maxilloturbinal (Curtis and Simmons, 2017; Curtis et al., 2020) (Figure 9). Curtis and Simmons (2017) and Curtis et al. (2020) reported that the maxilloturbinal forms two strand-shaped structures that project rostrally and enter the nasopharyngeal duct, referring to these structures as the lateral and medial strand of the maxilloturbinals (Curtis and Simmons, 2017; Curtis et al., 2020).

In our view, their “maxilloturbinal” is not the true maxilloturbinal (Figure 9). Our observations on fetal and adult specimens in *Rhinolophus* show that the true maxilloturbinal is reduced, and its caudal end may be partially ossified (Supplementary Figure 9). We cannot be certain that it is ossified as we observed using the iodine-based solution. Observing the histological sections provided by Curtis et al. (2020), we can see that the structure we believe to be the maxilloturbinal (Curtis et al., 2020 also identifies a part of this as the maxilloturbinal) is unossified. Other Rhinolophoidea members, *H. gentilis*, and *A. stoliczkanus* also show similar maxilloturbinal like *Rhinolophus* with reduced and partially ossified caudal end even in the adult (Supplementary Figures 5–8). Curtis et al. (2020) also show the histological sections of *Hipposideros lankadiva*, and what we see as maxilloturbinal is unossified. In *Hipposideros*, the identification of the maxilloturbinal is congruent among their and our studies. Nonetheless, they do not present a 3D reconstruction; thus, we cannot be definite.

The anatomical definition of the maxilloturbinal is described in Maier’s therian bauplan for the nasal capsule (Figure 1) (Maier, 1993a). Maier has demonstrated that the maxilloturbinal is continuous with the atrioturbinal, observing Primates, Prosimii (*Daubentonia madagascariensis*, *Galagoides demidoff*) and Platyrrhini (*Pithecia monachus*, *Saimiri sciureus*), and Scandentia (*Ptilocercus lowii*, *Tupaia belangeri*) (Maier, 1980, 2000; Maier and Ruf, 2014). The fact that the maxilloturbinal is continuous with the atrioturbinal appears to be the common pattern for therian mammals (Maier, 1993a). Following Maier’s bauplan, we identify the structure that is continuous with the atrioturbinal as the maxilloturbinal. The maxilloturbinal in Rhinolophoidea extends from the atrioturbinal in the early stage in *R. affinis* and *H. gentilis*, the mid stage in *R. pusillus*, and the late stage in *A. stoliczkanus* (Figures 5A,A′,F,F′, 6A,A′,G,G′ and Supplementary Figures 5–8).

So, what are the lateral and medial strands of the “maxilloturbinal” that project into the nasopharyngeal duct shown by Grosser (1900); Curtis and Simmons (2017), and Curtis et al. (2020)? In our view, their lateral strand of the maxilloturbinal is probably the ethmoturbinal pars anterior. In mammals, the first projection within the nasal capsule becomes ethmoturbinal I (Smith and Rossie, 2008). Our results show that presumptive initial fold first appears within the nasal capsule in the fetus (CS 15) of *R. pusillus* (Figures 8A,B). Then, the cartilaginous structure of ethmoturbinal I projects toward the initial fold in the fetus (CS 16) (Figures 8B,D).

The projecting cartilaginous structure of ethmoturbinal I pars anterior extends rostrally and turns slightly ventrally within the nasal capsule in the early stage of *R. pusillus* (Figures 5E,E′). The ethmoturbinal I pars anterior forms a hairpin-shaped structure with a distinctive curve during the mid stage to adult in *Rhinolophus* such that its tip extends caudally (Figures 5B–D′,E–H′). The position of the formed ethmoturbinal I pars anterior partly matches that of the lateral strand of the “maxilloturbinal” reported by Curtis and Simmons (2017) (Figure 9).

There are two possible interpretations regarding the “medial strand of the maxilloturbinal” of *Rhinolophus*. We cannot confirm whether it is part of the lamina horizontalis or part of the ethmoturbinal I pars anterior at this point. This is because in the region where the medial hairpin-shaped turbinal is formed, the boundary between the lamina horizontalis and the ethmoturbinal I is indistinctive, making the identification of the medial hairpin-shaped turbinate structure difficult. De Beer (1937) identified the lamina horizontalis as part of ethmoturbinal I. Our rationale is based on the observation of the topologies of turbinals during development, and further identification is unreasonable.

If the medial hairpin-shaped turbinate structure is the lamina horizontalis, it elongates medially in the early stage and extends ventrally and turns laterally from the mid to late stages (Figures 5A–C′,E–G′). In the adult, its apex extends caudally (Figures 5D,D′,H,H′). A comprehensive study on Rhinolophoidea is required to test this scenario.

If the medial hairpin-shaped turbinate structure is a part of ethmoturbinal I pars anterior, ethmoturbinal I pars anterior of *Rhinolophus* splits into rostral and the caudal parts. The rostral part of ethmoturbinal I pars anterior is the “lateral strand of the maxilloturbinals” in Curtis and Simmons (2017), and the caudal part is their “medial strand of the maxilloturbinals.” The caudal part of ethmoturbinal I pars anterior extends medially, then turns ventrally and continues toward the caudal direction from the early stage in *Rhinolophus* (Figures 5A,A′,E,E′). We found it as the medial hairpin-shaped turbinate structure within the nasopharyngeal duct in adult (Figures 5D,D′,H,H′, 9B and Supplementary Figures 5, 6).

We conclude that it is unlikely that the lateral hairpin-shaped turbinate structure is the ethmoturbinal I pars anterior and the medial hairpin-shaped turbinate structure is ethmoturbinal I pars posterior in Rhinolophidae. Ethmoturbinal I pars posterior is formed medially to ethmoturbinal I pars anterior and dorsally to the lamina horizontalis in Pteropodidae and in various non-bat mammals (Allen, 1882; Voit, 1909; Smith and Rossie, 2008; Maier and Ruf, 2014; Ruf et al., 2015; Ruf, 2020). Nonetheless, the medial hairpin-shaped turbinate structure is formed ventrally to the lamina horizontalis in *Rhinolophus.* The position is too far apart from where we would expect (where we can locate the ethmoturbinal I pars posterior in non-bat mammals).

Lateral and medial hairpin-shaped turbinate structures have not been reported from any previous study in other Rhinolophoidea including Hipposideridae (*Aselliscus tricuspidatus*, *Coleops frithii*, *Hipposideros armiger*, *Hipposideros fulvus*, *Hipposideros pratti*, and *Hipposideros speoris*), *Rhinopoma*, *Macroderma gigas*, *M. lyra*, and *Rhinonycteris aurantia* (Nelson et al., 2007; Smith et al., 2012; Curtis and Simmons, 2017; Curtis et al., 2020). However, as previous studies did not observe prenatal specimens and did not investigate both soft and hard parts of the turbinals, they did not rule out the possibility that the hairpin-shaped turbinate structure may be present in Hipposideridae (Curtis and Simmons, 2017). We found that ethmoturbinal I (the turbinal that forms the hairpin-shaped turbinal in Rhinolophidae) of Hipposideridae, *H. gentilis* and *A. stoliczkanus*, projects from the inner wall of the nasal capsule in the early stage and then enlarges toward the rostral direction after the early stage (Figure 6), confirming that these species do not form the hairpin-shaped turbinal. The lateral and medial hairpin-shaped turbinate structures that we identify as ethmoturbinal I pars anterior or the lamina horizontalis are certainly unique to Rhinolophidae.

### Character Evolution of Turbinals in Bats

**FIGURE 10:**
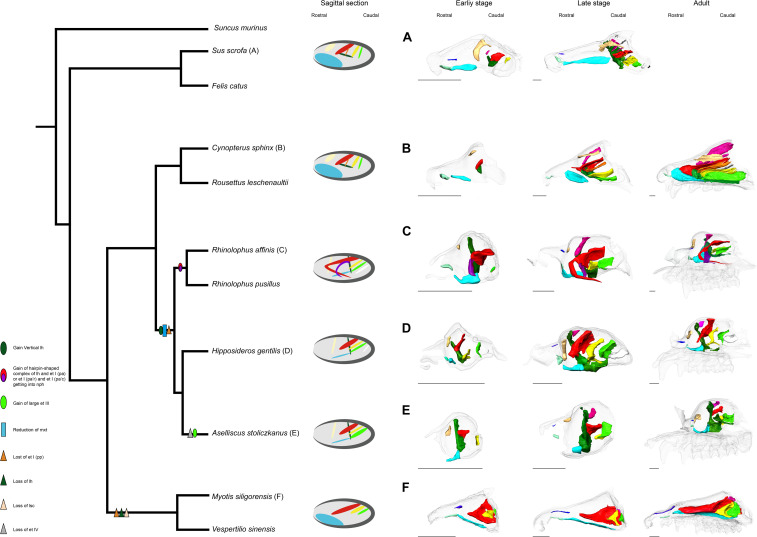
Inferred evolutionary history of the nasal structures. **(A)** *S. scrofa*; **(B)** *C. sphinx*; **(C)** *R. affinis*; **(D)** *H. gentilis*; **(E)** *A. stoliczkanus*; **(F)** *M. siligorensis*. Note that the inference for Yangochiroptera is based only on Vespertilionidae. ethmoturbinal I pars anterior (red); ethmoturbinal I pars posterior (orange); ethmoturbinal II (yellow); ethmoturbinal III (light green); ethmoturbinal IV (white); lamina horizontalis (green); lamina horizontalis or caudal part of ethmoturbinal I pars anterior (purple); lamina semicircularis (flesh color); maxilloturbinal (light blue).

The possible evolutionary scenario of the turbinal architecture in bats is summarized in Figure 10. Although several Eocene fossil bats including *Onychonycteris finneyi* have been reported (Simmons et al., 2008), their nasal capsule and nasal cavity are difficult to observe due to fossilization. Thus, we can only infer ancestral traits from extant species. Giannini et al. (2012) pointed out that the turbinate morphology of Pteropodidae is highly comparable to that of non-volant laurasiatherians. We found that Rhinolophoidea are characterized by the vertically stranding lamina horizontalis and the rostrally cartilaginous and caudally ossified maxilloturbinal (Figure 10). The lamina horizontalis does not separate the nasopharyngeal duct and the ethmoturbinal recess in our limited Yangochiroptera sample. Given these observations, the turbinate morphology seen in Pteropodidae is unlikely to be a result of convergence, and we assume that the bat common ancestor most probably had a turbinate morphology comparable to Pteropodidae and non-volant Laurasiatheria.

In both Pteropodidae and Rhinolophoidea, the lamina horizontalis separates the nasopharyngeal duct and ethmoturbinal recess. On the other hand, the maxilloturbinal is developed in Pteropodidae while it is reduced in Rhinolophoidea, and the orientation of the lamina horizontalis is horizontal in Pteropodidae while it is vertical in Rhinolophoidea. Ethmoturbinal I pars posterior is lost in Rhinolophoidea. It is reported that *R. aurantia* and *Triaenops persicus* have a small number of ethmoturbinals, while *M. lyra* has an increased number of ethomoturbinals (I–VII) (Smith et al., 2012; Curtis and Simmons, 2017). Thus, the diversification of ethmoturbinal numbers appears to characterize the Rhinolophoidea lineage.

Our results and previous literature on *M. schreibersii* (Fawcett, 1919; De Beer, 1937) and *M. myotis* (Frick, 1954) suggest that Yangochiroptera have lost ethmoturbinal I pars posterior (Figure 10). As noted earlier, ethmoturbinal I pars posterior is also lost in Rhinolophoidea. If the condition of the bat common ancestor was the same as Pteropodidae and non-volant laurasiatherians, the loss of ethmoturbinal I pars posterior in Yangochiroptera and Rhinolophoidea has occurred independently. Studies by Fawcett (1919); De Beer (1937), and Frick (1954), and ours suggest that oral-emitting Yangochiroptera (*M. schreibersii*, *M. myotis*, *M. siligorensis*, and *V. sinensis*) lack the lamina horizontalis, which separates the nasopharyngeal duct and ethmoturbinal recess in Yinpterochiroptera. On the other hand, nasal-emitting Yangochiroptera such as Phyllostomidae possess the structure that separates the nasopharyngeal duct and ethmoturbinal recess (Bhatnagar and Kallen, 1974a; Kämper and Schmidt, 1977; Yohe et al., 2018). This raises a new question: whether the structure that separates the nasopharyngeal duct and ethmoturbinal recess in Yinpterochiroptera and Phyllostomidae is homologous or not. In Yinpterochiroptera, the separation between the nasopharyngeal duct and ethmoturbinal recess in the rostral part of the nasal cavity is achieved by the lamina horizontalis. In contrast, the separation is achieved by the complex structure of ethmoturbinals in Phyllostomidae (Bhatnagar and Kallen, 1974a; Kämper and Schmidt, 1977; Yohe et al., 2018). It may be possible that the separation occurred secondarily in Phyllostomidae after the lamina horizontalis was lost in the common ancestor of Yangochiroptera, although this needs to be examined through observations on prenatal specimens of Phyllostomidae.

Using fetal specimens of various bat species, we have described the detailed 3D development of the nasal turbinals in bats and mostly resolved the confused homology of turbinals, though there are still some questions regarding the lamina horizontalis in Yangochiroptera and rostral turbinate structures of bats. Our study emphasizes the importance of studying prenatal anatomy and observing 3D structures of turbinals to address its homology problems. However, our study did not include members of Yangochiroptera from the New World, such as Phyllostomidae, Mollossidae, Emballonuridae, and Nycteridae. Currently, whether laryngeal echolocation has a single origin in bats or evolved multiple times independently is still disputed (Teeling et al., 2005; Nojiri et al., in press). The character states of the nasal turbinals of the common ancestor and how nasal turbinals have evolved with the evolution of laryngeal echolocation are still unknown. We envision future studies on the prenatal anatomy of bats to clarify the picture of their turbinal evolution and also solve the remaining problems associated with the homology of this complex structure among Laurasiatheria.

## Conclusion

Using diceCT imaging, we comparatively described the 3D prenatal development of the nasal cavity in eight bat species of Yangochiroptera and Yinpterochiroptera and three species of non-volant Laurasiatheria. By observing multiple stages of nasal development, we solved the confused turbinate homology among bats and clarified the evolutionary history of the nasal turbinals in bats. We found that the strand-shaped structure of the “maxilloturbinal” of Rhinolophidae in Grosser (1900); Curtis and Simmons (2017), and Curtis et al. (2020) is not the true maxilloturbinal. We conclude that the “maxilloturbinal” of Grosser (1900); Curtis and Simmons (2017), and Curtis et al. (2020) is actually the complex of a part of the lamina horizontalis and ethmoturbinal I pars anterior or the rostral and caudal splitting parts of ethmoturbinal I pars anterior. The true maxilloturbinal is an undeveloped structure with a cartilaginous rostral part and an ossified caudal part, even in the adult. We found that the turbinate structures are principally comparable between Laurasiatheria and Pteropodidae, suggesting that Pteropodidae retain the basic condition of Laurasiatheria.

Pteropodidae exhibit an enlarged ethmoturbinal recess similar to non-volant mammals with a keen olfactory sense. The ethmoturbinal recess is significantly smaller in Rhinolophoidea compared with its closely related Pteropodidae, suggesting its reduced capability of olfaction. The lack of separation between the nasopharyngeal duct and ethmoturbinal recess in oral-emitting Yangochiroptera may indicate they are not well specialized for odorant deposition along the olfactory epithelium.

In both Pteropodidae and Rhinolophoidea, the lamina horizontalis separates the nasopharyngeal duct and ethmoturbinal recess. The maxilloturbinal is well developed in Pteropodidae, while it is reduced in Rhinolophoidea. The orientation of the lamina horizontalis is horizontal in Pteropodidae, while it is vertical in Rhinolophoidea. Rhinolophoidea are characterized by a well-developed vertically standing lamina horizontalis. It also acquired a rostrally cartilaginous and caudally ossified maxilloturbinal. The absence of ethmoturbinal I pars posterior in Yangochiroptera and Rhinolophoidea has occurred independently by convergent evolution. The separation of the nasopharyngeal duct and ethmoturbinal recess is found in Yinpterpchiroptera and Phyllostomidae, but not in oral-emitting Yangochiroptera. Whether the separation structure found in Yinpterpchiroptera and Phyllostomidae is homologous or it has evolved secondarily in Phyllostomidae should be tested in future studies.

## Data Availability Statement

The raw data supporting the conclusions of this article are available from the authors upon request.

## Ethics Statement

The animal study was reviewed and approved by Institute of Ecology and Biological Resources, Vietnam Academy of Science and Technology.

## Author Contributions

All authors listed have made a substantial, direct and intellectual contribution to the work, and approved it for publication.

## Conflict of Interest

The authors declare that the research was conducted in the absence of any commercial or financial relationships that could be construed as a potential conflict of interest.
