# Supplementary material for: On the Embryonic Development of the Nasal Turbinals and Their Homology in Bats
Source: Front Cell Dev Biol. 2021 Mar 23;9:613545. doi: 10.3389/fcell.2021.613545 (PMC8021794; doi:10.3389/fcell.2021.613545)
Supplement: Supplementary file 1 [file Data_Sheet_1.PDF]

Supplementary Material, Figure S1

Virtual model of cranial bone (yellow) and cartilaginous nasal capsule (blue) of *Miniopterus fuliginosus* in the late stage (CS22). **(A, D)** Dorsal view of the right side; **(B, E)** Parasagittal section showing lateral wall of the right nasal capsule; **(C, F)** Lateral view of the left side; scale = 1 mm. Abbreviations: fr = frontal; pm = premaxilla; man = mandible; mx = maxilla; nc = nasal capsule; pa = parietal

Supplementary Material, Figure S2

Coronal section of μCT images of *Suncus murinus*, *Sus scrofa*, and *Felis catus* from anterior to posterior.

(A–F) show approximate location of section through the nasal capsule in each species. **(A-1–5)** Mid stage fetus of *S. murinus.* **(B-1–5)** Late stage fetus of *S. murinus.* **(C-1–5)** Mid stage fetus of *S. scrofa.* **(D-1–5)** Late stage fetus of *S. scrofa*. **(E-1–5)** Mid stage fetus of *F. catus*. (**F-1–5)** Late stage fetus of *F. catus*. Scale bars, 1mm. Abbreviations: at = atrioturbinal; et I (pa) = ethmoturbinal I pars anterior; et I (pp) = ethmoturbinal I pars posterior; et II–IV = ethmoturbinal II–IV; ft = frontoturbinal; mxt = maxilloturbinal; lh = lamina horizontalis; lsc = lamina semicircularis; nt = nasoturbinal

Supplementary Material, Figure S3

Coronal section of μCT images of *Cynopterus sphinx.*

(A–D) show approximate location of section through the nasal capsule or nasal cavity. **(A-1–4)** Early stage fetus. **(B-1–5)** Mid stage fetus. **(C-1–5)** Late stage fetus. **(D-1–5)** Adult. Scale bars, 1mm. Abbreviations: at = atrioturbinal; et I (pa) = ethmoturbinal I pars anterior; et I (pp) = ethmoturbinal I pars posterior; et II–IV = ethmoturbinal II–IV; ft = frontoturbinal; if = initial fold; mxt = maxilloturbinal; lh = lamina horizontalis; lsc = lamina semicircularis; nt = nasoturbinal

Supplementary Material, Figure S4

Coronal section of μCT images of *Rousettus leschenaultii.*

(A–D) show approximate location of section through the nasal capsule or nasal cavity. **(A-1–4)** Early stage fetus. **(B-1–5)** Mid stage fetus. **(C-1–5)** Late stage fetus. **(D-1–5)** Adult. Scale bars, 1mm. Abbreviations: at = atrioturbinal; et I (pa) = ethmoturbinal I pars anterior; et I (pp) = ethmoturbinal I pars posterior; et II–IV = ethmoturbinal II–IV; ft = frontoturbinal; if = initial fold; mxt = maxilloturbinal; lh = lamina horizontalis; lsc = lamina semicircularis; nt = nasoturbinal

Supplementary Material, Figure S5
Coronal section of µCT images of *Rhinolophus affinis.*
(A–D) show approximate location of section through the nasal capsule or nasal cavity. **(A-1–4)** Early stage fetus. **(B-1–4)** Mid stage fetus. **(C-1–4)** Late stage fetus. **(D-1–4)** Adult. Scale bars, 1mm. Abbreviations: at = atrioturbinal; et I (pa) = ethmoturbinal I pars anterior; et I (pa'c) = caudal part of ethmoturbinal I pars anterior; et I (pp) = ethmoturbinal I pars posterior; et II–III = ethmoturbinal II–III; ft = frontoturbinal; mxt = maxilloturbinal; lh = lamina horizontalis; lsc = lamina semicircularis; nt = nasoturbinal

Supplementary Material, Figure S6
Coronal section of µCT images of *Rhinolophus pusillus.*
(A–D) show approximate location of section through the nasal capsule or nasal cavity. **(A-1–4)** Early stage fetus. **(B-1–4)** Mid stage fetus. **(C-1–4)** Late stage fetus. **(D-1–4)** Adult. Scale bars, 1mm. Abbreviations: at = atrioturbinal; et I (pa) = ethmoturbinal I pars anterior; et I (pa'c) = caudal part of ethmoturbinal I pars anterior; et I (pp) = ethmoturbinal I pars posterior; et II–III = ethmoturbinal II–III; ft = frontoturbinal; mxt = maxilloturbinal; lh = lamina horizontalis; lsc = lamina semicircularis; nt = nasoturbinal

Supplementary Material, Figure S7
Coronal section of µCT images of *Hipposideros gentilis.*
(A–D) show approximate location of section through the nasal capsule or nasal cavity. **(A-1–5)** Early stage fetus. **(B-1–5)** Mid stage fetus. **(C-1–5)** Late stage fetus. **(D-1–5)** Adult. Scale bars, 1mm. Abbreviations: at = atrioturbinal; et I (pa) = ethmoturbinal I pars anterior; et I (pp) = ethmoturbinal I pars posterior; et II–IV = ethmoturbinal II–IV; ft = frontoturbinal; mxt = maxilloturbinal; lh = lamina horizontalis; lsc = lamina semicircularis; nt = nasoturbinal

Supplementary Material, Figure S8
Coronal section of µCT images of *Aselliscus stoliczkanus.*
(A–D) show approximate location of section through the nasal capsule or nasal cavity. **(A-1–4)** Early stage fetus. **(B-1–4)** Mid stage fetus. **(C-1–5)** Late stage fetus. **(D-1–5)** Adult. Scale bars, 1mm. Abbreviations: at = atrioturbinal; et I (pa) = ethmoturbinal I pars anterior; et I (pp) = ethmoturbinal I pars posterior; et II–III = ethmoturbinal II–III; ft = frontoturbinal; mxt = maxilloturbinal; lh = lamina horizontalis; lsc = lamina semicircularis; nt = nasoturbinal

Supplementary Material, Figure S9

Rostrocaudal sequence of adult *Rhinolophus pusillus* from its rostral end showing the ossified maxilloturbinal (light blue).

(a–c) Maxilloturbinal remains cartilaginous while ethmoturbinal I pars anterior and lamina horizontalis are ossified. **(d–f)** Bony structure can be seen in the cartilaginous maxilloturbinal. Scale bars, 1mm. Abbreviations: et I (pa) = ethmoturbinal I pars anterior; et I (pa'c) = caudal part of ethmoturbinal I pars anterior; mxt = maxilloturbinal; lh = lamina horizontalis; os mxt = ossified maxilloturbinal

Supplementary Material, Figure S10

Coronal section of µCT images of *Myotis siligorensis.*

(A–D) show approximate location of section through the nasal capsule or nasal cavity. **(A-1–4)** Early stage fetus. **(B-1–4)** Mid stage fetus. **(C-1–4)** Late stage fetus. **(D-1–4)** Adult. Scale bars, 1mm. Abbreviations: at = atrioturbinal; et I = ethmoturbinal I; et II–III = ethmoturbinal II–III; ft = frontoturbinal; mxt = maxilloturbinal; lh = lamina horizontalis; nt = nasoturbinal; lsc = lamina semicircularis

Supplementary Material, Figure S11

Coronal section of µCT images of *Vespertilio sinensis.*

(A–D) show approximate location of section through the nasal capsule or nasal cavity. **(A-1–4)** Early stage fetus. **(B-1–4)** Mid stage fetus. **(C-1–4)** Late stage fetus. **(D-1–4)** Adult. Scale bars, 1mm. Abbreviations: at = atrioturbinal; et I = ethmoturbinal I; et II–III = ethmoturbinal II–III; ft = frontoturbinal; mxt = maxilloturbinal; lh = lamina horizontalis; nt = nasoturbinal; lsc = lamina semicircularis

Supplementary Material, Figure S12

Schematic of mid-sagittal section of nasal cavity of *Epomophorus gambianus* by Allen (1882), Plate V, Figure 1. **(A)** descriptions by Allen. Abbreviations (modified in this study): ec I–IV = ectoturbinal I–IV; mxt = maxilloturbinal. **(B)** revised descriptions by this study. Abbreviations (modified in this study): et I (pa) = ethmoturbinal I pars anterior; et I (pp) = ethmoturbinal I pars posterior; et II, III = ethmoturbinal II, III; mxt = maxilloturbinal.

Supplementary Material, Figure S13

Schematic of the frontal section of the posterior nasal structure of *Rhinolophus ferrumequinum* by Grosser (1900), Figure 17. Abbreviations (modified in this study): et

III = ethmoturbinal III; et V = ethmoturbinal V; mxt = maxilloturbinal; nph = nasopharyngeal duct.
