# Supplementary material for: On the Embryonic Development of the Nasal Turbinals and Their Homology in Bats
Source: Front Cell Dev Biol. 2021 Mar 23;9:613545. doi: 10.3389/fcell.2021.613545 (PMC8021794; doi:10.3389/fcell.2021.613545)
Supplement: Supplementary file 2 [file Data_Sheet_2.PDF]

Supplementary Table 1. Distribution, the number of turbinals and laminas, scan resolution, and specimen number

Species	Stage		CRL (mm)	Maxilloturbinal	Lamina semicircularis	Medial projection of lateral nasal wall	Frontoturbinal	Ethmoturbinals	Ethmoturbinal I posterior part	Resolution (isotropic voxel size in mm)	Specimen ID
Suncus murinus	early	gestation day 29	19.8	X	X	X	2	3	X	0.015	UMUT_Suncus_KI02
	late	postnatal day1	26.0	X	X	X	2	3	X	0.011	UMUT_Suncus_P1day_CRL26
Sus scrofa	early	gestation day c.a. 28	17.9	X	X	X	1	2	-	0.011	UMUT_Pig_K013_KI_CRL18
	late	gestation day c.a. 40	42.4	X	X	X	3	4	X	0.016	UMUT_Pig_K76_KI_CRL42
Felis catus	early	gestation day 38	58.8	X	X	X	3	3	X	0.018	UMUT_Cat_K025_KI_CRL59
	late	gestation day 49	99.5	X	X	X	3	3	X	0.035	UMUT_Cat_K025_KI_CRL100
Cynopterus sphinx	early	CS 18	10.9	X	X	X	-	1	-	0.008	VN18-11
	mid	CS 19	14.6	X	X	X	1	3	X	0.011	VN18-03
	late	CS 22	22.2	X	X	X	1	3	X	0.015	VN18-72
	adult	adult	NA	X	X	X	1	3	X	0.034	BB06
Rousettus leschenaultii	early	CS 18	10.5	X	X	X	-	1	-	0.009	VN17-366
	mid	CS 19	16.0	X	X	X	1	3	X	0.012	VN17-357
	late	CS 23	24.2	X	X	X	1	3	X	0.016	VN18-45
	adult	adult	NA	X	X	X	1	3	X	0.031	UMUT_KI19-001_sl41
Rhinolophus affinis	early	CS 18	8.9	X	X	X	-	2	-	0.010	VN17-146
	mid	CS 19	15.6	X	X	X	1	4	-	0.013	QN013
	late	CS 22	20.5	X	X	X	1	4	-	0.011	BNB2016.0317.3
	adult	adult	NA	X	X	X	1	4	-	0.022	VN11-188
Rhinolophus pusillus	early	CS 15	7.4	-	-	-	-	-	-	0.008	170323DF01
	early	CS 16	9.6	X	-	X	-	3	-	0.010	BNB20160317.8
	mid	CS 19	13.5	X	X	X	-	4	-	0.014	QN41
	late	CS 22	19.5	X	X	X	-	4	-	0.012	B1604134
	adult	adult	NA	X	X	X	1	4	-	0.013	VTTU15-005
Hipposideros gentilis	early	CS 18	9.3	X	X	X	-	4	-	0.015	B170324DF-14
	mid	CS 19	14.7	X	X	X	-	4	-	0.013	VN17-190
	late	CS 23	22.0	X	X	X	1	4	-	0.013	XL-2016-34
	adult	adult	NA	X	X	X	1	4	-	0.016	VN17-364
Aselliscus stoliczkanus	early	CS 18	9.3	X	X	X	-	2	-	0.010	VN17-112
	mid	CS 19	14.9	X	X	X	-	3	-	0.011	XL2016-05
	late	CS 22	21.1	X	X	X	1	3	-	0.011	XL2016-09
	adult	adult	NA	X	X	X	1	3	-	0.012	VTTU15-013
Myotis siligorensis	early	CS 18	10.8	X	-	-	1	2	-	0.010	VN-17-188
	mid	CS 19	13.0	X	-	-	1	3	-	0.008	VN17-233
	late	CS 23	19.1	X	-	-	1	3	-	0.011	Lc-44
	adult	adult	NA	X	-	-	1	3	-	0.013	VTTU14-0148
Vespertilio sinensis	early	CS 18	9.2	X	-	-	1	2	-	0.009	NT0032
	mid	CS 19	12.4	X	-	-	1	3	-	0.010	NT0039
	late	CS 22	23.9	X	-	-	1	3	-	0.019	NT0046
	adult	adult	NA	X	-	-	1	3	-	0.019	KPM2447
